# Induction of Viral Mimicry Upon Loss of DHX9 and ADAR1 in Breast Cancer Cells

**DOI:** 10.1158/2767-9764.CRC-23-0488

**Published:** 2024-04-04

**Authors:** Kyle A. Cottrell, Sua Ryu, Jackson R. Pierce, Luisangely Soto Torres, Holly E. Bohlin, Angela M. Schab, Jason D. Weber

**Affiliations:** 1 Department of Medicine, Division of Molecular Oncology, Washington University School of Medicine, St. Louis, Missouri.; 2 ICCE Institute, Washington University School of Medicine, St. Louis, Missouri.; 3 Department of Biochemistry, Purdue University, West Lafayette, Indiana.; 4 Department of Cell Biology and Physiology, Washington University School of Medicine, St. Louis, Missouri.; 5 Department of Biology, Siteman Cancer Center, St. Louis, Missouri.

## Abstract

Detection of viral double-stranded RNA (dsRNA) is an important component of innate immunity. However, many endogenous RNAs containing double-stranded regions can be misrecognized and activate innate immunity. The IFN-inducible ADAR1-p150 suppresses dsRNA sensing, an essential function for adenosine deaminase acting on RNA 1 (ADAR1) in many cancers, including breast. Although ADAR1-p150 has been well established in this role, the functions of the constitutively expressed ADAR1-p110 isoform are less understood. We used proximity labeling to identify putative ADAR1-p110–interacting proteins in breast cancer cell lines. Of the proteins identified, the RNA helicase DHX9 was of particular interest. Knockdown of DHX9 in ADAR1-dependent cell lines caused cell death and activation of the dsRNA sensor PKR. In ADAR1-independent cell lines, combined knockdown of DHX9 and ADAR1, but neither alone, caused activation of multiple dsRNA sensing pathways leading to a viral mimicry phenotype. Together, these results reveal an important role for DHX9 in suppressing dsRNA sensing by multiple pathways.

**Significance:**

These findings implicate DHX9 as a suppressor of dsRNA sensing. In some cell lines, loss of DHX9 alone is sufficient to cause activation of dsRNA sensing pathways, while in other cell lines DHX9 functions redundantly with ADAR1 to suppress pathway activation.

## Introduction

RNA editing enhances protein diversity and modulates multiple aspects of RNA metabolism (1–3). A-to-I editing is carried out by adenosine deaminase acting on RNA 1 (ADAR1), an RNA editase that binds double-stranded RNA (dsRNA) and converts adenosines to inosines (4, 5). The main domains of ADAR1 include the Z-DNA binding domains (ZBD), the dsRNA binding domains (dsRBD), and the deaminase domain (6, 7). There are two isoforms, p110 and p150, produced by alternative transcriptional start sites (8). They share the same deaminase domain, dsRBDs, and a ZBD, but exhibit a distinct subcellular localization (9, 10). ADAR1-p150 is predominantly cytoplasmic, whereas ADAR1-p110 is nuclear localized (9, 11).

An important role for ADAR1 is to suppress dsRNA sensing (12–15). Many endogenously encoded RNAs can form large double-stranded regions, often through base pairing between inverted Alu elements (16). ADAR1 edits the majority of human genes, with most editing occurring within inverted Alu repeats (3, 4). By binding, editing, and thus altering the structure of dsRNA, ADAR1 suppresses the detection of dsRNA by various cytoplasmic sensors such as MDA5, RIG-I, and PKR (12, 13, 15, 17, 18). These RNA sensors are part of innate immunity against viral infections (16, 19–21). Thus, ADAR1 prevents the activation of innate immunity pathways by endogenous immunogenic RNAs (16, 20, 22). Some mutations of ADAR1 in humans cause inappropriate dsRNA sensing and activation of the type I IFN (IFN-I) pathway which manifests as the interferonopathy Aicardi–Goutières syndrome (23).

The cell-intrinsic antiviral response against foreign dsRNA—or misrecognized endogenous dsRNA—involves multiple pathways: (i) Recognition of dsRNA by MDA5 or RIG-I results in the activation of IFN-I signaling (16, 20, 22). (ii) Activation of the dsRNA-binding kinase PKR triggers translational shutdown by phosphorylation of the translation initiation factor eIF2α (12). (iii) Detection of dsRNA by OAS proteins activates RNase L, which carries out nonspecific cleavage of RNA and triggers cell death (24). There is significant cross-talk between the three pathways, and the aforementioned dsRNA sensors are transcriptionally controlled by IFN-I, known as interferon-stimulated genes (ISG; refs. 25, 26). The ADAR1-p150 isoform is itself an ISG and is the isoform responsible for suppressing the activation of dsRNA sensors (8, 10, 14, 27). ADAR1-p110, however, is constitutively expressed, though its functions are less established (28, 29).

Because IFN-I signaling is often cytotoxic and antiproliferative, ADAR1’s ability to suppress IFN-I signaling was shown to exert protumor effects (22, 30, 31). As such, ADAR1 has been proposed as a potential therapeutic target for various cancers, including breast cancer (30, 32–34). ADAR1 mRNA expression is elevated in breast cancer and is correlated with a poor prognosis (30, 35). Furthermore, ADAR1 is essential for a subset of breast cancer cells overrepresented by triple-negative breast cancer (TNBC; ref. 35). Known as “ADAR1-dependency,” depletion of ADAR1-p150 leads to IFN-I signaling and global translational repression in cells that are sensitive to depletion of ADAR1 (34, 35). What makes cancer cells sensitive or refractory to ADAR1 loss is not yet determined (35).

ADAR1 interacts with numerous RNA-binding proteins including RNA helicases, transcription machinery, and DNA repair proteins (29, 36–39). The influence of ADAR1-interacting proteins on A-to-I editing has previously been reported (37, 40–43). In this study, we evaluated components of the ADAR1 interactome in breast cancer cells and identified the RNA helicase DHX9 as a redundant suppressor of immunogenic dsRNA in ADAR1-independent breast cancer cells. We demonstrate that codepletion of ADAR1 and DHX9 is sufficient to trigger a viral mimicry phenotype in ADAR1-independent cells.

## Materials and Methods

### Cell Culture

Breast cancer cell lines (MCF-7 (RRID:CVCL_0031), SK-BR-3 (RRID:CVCL_0033), BT-549 (RRID: CVCL_1092), MDA-MB-231 (RRID: CVCL_0062), HCC1806 (RRID: CVCL_1258), MDA-MB-468 (RRID: CVCL_0063), and 293T (RRID: CVCL_0063) were obtained from ATCC which used short tandem repeat profiling to authenticate the cell lines. All cell lines were cultured in DMEM (Hyclone) with 10% FBS (BioTechne), 2 mmol/L glutamine (Hyclone), 0.1 mmol/L nonessential amino acids (Hyclone), and 1 mmol/L sodium pyruvate (Hyclone). *Mycoplasma* testing was performed by a PCR-based method with the most recent test in October 2023. Experiments described here were performed within 30 passages after thawing cells.

### Viral Production and Transduction

Lentivirus was produced by Turbo DNAfection 3000 or LipoFexin (Lamda Biotech) transfection of 293T cells with pCMV-VSV-G, pCMV-ΔR8.2, and the appropriate plasmid for expression of genes of interest or short hairpin RNAs (shRNA). Virus was harvested 48 hours posttransfection. Cells were transduced with lentivirus for 16 hours in the presence of 10 µg/mL protamine sulfate (Sigma-Aldrich). The cells were selected with puromycin at 2 µg/mL (Sigma-Aldrich), 150 µg/mL hygromycin (Invitrogen) or 10 µg/mL Blasticidin (Invitrogen).

### Plasmids

APEX2 was PCR amplified from pcDNA3-APEX2-NES, a kind gift from the laboratory of Kendall Blumer at Washington University in St. Louis (St. Louis, MO). ADAR1-p110 was PCR amplified from pLVX-p110-ADAR1 described previously (35). APEX2 and p110 were cloned into pLVX-IRES-puro (Takara, 632183) via a series of restriction enzyme digests and ligations. The final plasmids pLVX-3xFLAG-APEX2 and pLVX-3xFLAG-APEX2-linker-p110 were confirmed by digestion and sequencing. The linker consists of three repeats of Gly-Gly-Gly-Gly-Ser. Lentiviral shRNA constructs in the pLKO.1-puro vector were purchased as glycerol stocks from Millipore Sigma. For shADAR1, the shRNA was subcloned into pLKO.1-hygro, a gift from Bob Weinberg (Addgene, #24150). The sequences for the shRNA-scramble (shSCR) and shRNA-ADAR1 (shADAR1) were described and validated previously (35). The sequences for shRNAs against DHX9 and DDX17 are in Supplementary Table S1. For sgDHX9, oligos encoding the sgRNAs (Supplementary Table S1) were cloned into lentiCRISPR v2, a gift from Feng Zhang (Addgene, #52961; ref. 44). Overexpression constructs for DHX9 and ADAR were generated by PCR amplification and ligation into pLV-EF1a-IRES-Blast vector, a gift from Tobias Meyer (Addgene, #85133; ref. 45). For DHX9 overexpression, wobble mutants were made to reduce shRNA targeting. Mutagenesis primers for DHX9 K417R and shRNA-resistant codons [designed using the Synonymous Mutation Generator (46)] are included in Supplementary Table S1. The DHX9-dsRBD-EGFP construct was generated by digestion of pLV-EF1-DHX9 with SpeI and EcoRI and ligation of EGFP in place of the 3′ portion of DHX9. The resulting construct codes for the first 344 amino acids of DHX9 fused to EGFP.

### Immunoblot

Cell pellets were lysed and sonicated in RIPA Buffer [50 mmol/L Tris pH 7.4 (Ambion), 150 mmol/L NaCl (Ambion), 1% Triton X-100 (Sigma-Aldrich), 0.1% SDS (Promega), and 0.5% sodium deoxycholate (Sigma-Aldrich)] with 1x HALT Protease Inhibitor (Pierce). Protein was quantified using the DC Assay kit (Bio-Rad) and diluted in SDS Sample Buffer (125 mmol/L Tris pH 6.8, 30% glycerol, 10% SDS, 0.012% bromophenol blue) prior to denaturation by heating to 95°C for 7 minutes. A total of 30 µg of protein lysate were resolved on 4%–12% TGX Acrylamide Stain-Free gels (Bio-Rad). Stain-Free gels were imaged prior to transfer to polyvinylidene difluoride membrane (Millipore or Bio-Rad) by TransBlot Turbo (Bio-Rad). The blots were then probed with the appropriate primary antibodies: ADAR1 (Santa Cruz Biotechnology, catalog no. sc-73408, RRID:AB_2222767; Bethyl, catalog no. A303-883A, RRID:AB_2620233), DDX17 (Thermo Fisher Scientific, catalog no. PA5-84585, RRID:AB_2791736), DHX9 (Bethyl, catalog no. A300-855A, RRID:AB_609442), DDX54 (Novus, catalog no. NB100-60678, RRID:AB_921120), eIF2a (Abcam, catalog no. ab5369, RRID:AB_304838), eIF2a-Ser-51-P (Abcam, catalog no. ab32157, RRID:AB_732117), Fibrillarin (Santa Cruz Biotechnology, catalog no. sc-25397, RRID:AB_640513), beta-tubulin (Abcam, catalog no. ab6046, RRID:AB_2210370), ISG15 (Santa Cruz Biotechnology, catalog no. sc-166755, RRID:AB_2126308), cleaved PARP (Cell Signaling Technology, catalog no. 9541, RRID:AB_331426), PKR (Cell Signaling Technology, catalog no. 3072, RRID:AB_2277600), PKR Thr-446-P (Abcam, catalog no. ab32036, RRID:AB_777310), MDA5 (Cell Signaling Technology, catalog no. 5321S, RRID:AB_10694490), MAVS (Cell Signaling Technology, catalog no. 3993S, RRID:AB_823565), ADAR1-p150 (Abcam, catalog no. ab126745, RRID:AB_11145661). Primary antibodies were detected with horseradish peroxidase (HRP)-conjugated secondary antibodies (Jackson ImmunoResearch) and detection was carried out with Clarity Western enhanced chemiluminescence (ECL) Substrate (Bio-Rad). Densitometry was performed using Image Lab (Bio-Rad). Band intensity was normalized to total protein measured by imaging of the Stain-Free gel.

### Proximity Labeling by APEX2

SK-BR-3, MCF-7, or MDA-MB-231 cells expressing pLVX-puro-FLAG-APEX2 or pLVX-puro-FLAG-APEX2-ADAR1p110 were grown to approximately 80% confluency in a 15 cm dish. Quencher solution [10 mmol/L sodium azide, 10 mmol/L sodium ascorbate, and 5 mmol/L Trolox (Sigma-Aldrich) in 1X PBS] was prepared at 1X and 2X concentrations. Prior to labeling, cells were incubated in 10 mL of culture media containing 500 µmol/L biotin phenol (Toronto Research Chemicals, B397770) for 30 minutes at 37°C. Next, hydrogen peroxide was added to the cells at 1 mmol/L and incubated at room temperature for 1 minute. Immediately one volume of 2X quencher solution was added to the cells to stop labeling. The cells were washed twice with 1X quencher solution. Cells were harvested by scraping in 1X quencher solution and lysed in RIPA buffer containing 10 mmol/L sodium azide, 10 mmol/L sodium ascorbate, 5 mmol/L Trolox, and 1x HALT. Biotin labeling was verified by immunoblotting with HRP-streptavidin (Abcam, ab7403). Biotinylated proteins were purified using streptavidin magnetic beads (Thermo Fisher Scientific, 88816). Streptavidin magnetic beads were washed twice with RIPA containing HALT and quenching agents. The lysate from above was incubated with the beads for 1 hour at room temperature. The beads were washed in the following order: once with RIPA containing HALT and quenching agents, once with RIPA, once with 1 mol/L KCl, once with 2 mol/L urea pH 8.0, twice in RIPA, and once with water. Elution was performed in 1X SDS-Sample buffer by heating at 95°C for 10 minutes. The eluate was analyzed by LC/MS-MS, see below.

### Mass Spectrometry

LC/MS-MS was performed by MSBioWorks. The eluates from the streptavidin pulldown above were processed by SDS-PAGE using 10% Bis-Tris NuPage Mini-gel (Invitrogen) with the MES buffer system. The gel was run 2 cm. The mobility region was excised and processed by in-gel digestion with trypsin using a robot (ProGest, DigiLab). For the trypsin digestion, the gel slices were washed with 25 mmol/L ammonium bicarbonate followed by acetonitrile. The samples were reduced with 10 mmol/L dithiothreitol at 60°C followed by alkylation with 50 mmol/L iodoacetamide at room temperature. Subsequently proteins were digested with trypsin (Promega) at 37°C for 4 hours. The trypsin digestion was quenched with formic acid and the supernatant was analyzed directly without further processing. The digested sample was analyzed by nano-LC/MS-MS with a Waters M-Class HPLC system interfaced to a Thermo Fisher Scientific Fusion Lumos mass spectrometer. Peptides were loaded on a trapping column and eluted over a 75 µm analytic column at 350 nL/minute; both columns were packed with Luna C18 resin (Phenomenex). The mass spectrometer was operated in data-dependent mode, with the Orbitrap operating at 60,000 FWHM and 15,000 FWHM for mass spectrometry (MS) and MS-MS, respectively. APD was enabled and the instrument was run with a 3-second cycle for MS and MS-MS. Five hours of instrument time was used for the analysis of each sample.

### Analysis of MS Data

Data were searched using a local copy of Mascot (Matrix Science, RRID:SCR_014322) with the following parameters: Enzyme – Trypsin/P; Database – SwissProt Human (concatenated forward and reverse plus common contaminants); Fixed modification – Carbamidomethyl; Variable modifications – Oxidation; Acetyl; Pyro-Glu; Deamidation; Mass values – Monoisotopic; Peptide Mass Tolerance – 10 ppm; Fragment Mass Tolerance – 0.02 Da; Max Missed Cleavages – 2. Mascot DAT files were parsed into Scaffold (Proteome Software, RRID:SCR_014321) for validation, filtering and to create a nonredundant list per sample. Data were filtered using a 1% protein and peptide FDR and requiring at least two unique peptides per protein. Fold change of protein abundance was determined by DESeq2 (RRID:SCR_015687) using spectral counts (see Data Availability below for scripts). Overrepresentation analysis was performed using “enrichR” (RRID:SCR_001575; ref. 47) in R (RRID:SCR_001905). The cutoff used for enrichment for the overrepresentation analysis was an FDR <0.05 and a log_2_ fold change of >0.5.

### Immunoprecipitation

Cell lysates prepared in RIPA with 1X HALT. RNaseOUT (Thermo Fisher Scientific) RNase inhibitor was added to the lysis buffer at 0.5 U/µL when RNase A was not used. A total of 1 mg of protein lysate was mixed with 2–10 µg of IgG or specific antibody overnight at 4°C with rotation. For samples treated with RNase A, 20 µg of RNase A (Invitrogen) or 20 U RNase III with MnCl_2_ added to 20 nmol/L (NEB), was added to the lysate during overnight mixing with the antibody. Protein G Dynabeads (Thermo Fisher Scientific, 25 µL per sample) were prepared by washing twice in the lysis buffer. Prepared beads were mixed with lysates for 30 minutes at 4°C with rotation. Supernatants were collected and beads were washed three times in the lysis buffer, and eluted by mixing the beads in SDS sample buffer and incubating at 95°C for 7 minutes. Antibodies: Rabbit IgG (Jackson ImmunoResearch, 011-000-003), Mouse IgG (Jackson ImmunoResearch Labs, catalog no. 015-000-003, RRID:AB_2337188), DHX9 (Bethyl, catalog no. A300-855A, RRID:AB_609442), PARP (Cell Signaling Technology, catalog no. 9532, RRID:AB_659884), XRN2 (Novus, catalog no. NB100-57541, RRID:AB_2288770), DDX54 (Novus, catalog no. NB100-60678, RRID:AB_921120), DDX17 (Thermo Fisher Scientific, catalog no. PA5-84585, RRID:AB_2791736).

### Immunofluorescence

Cells were plated on glass coverslips (Corning) 2 days prior to fixation for immunofluorescence. Cells were washed in PBS prior to fixation with 4% paraformaldehyde (Thermo Fisher Scientific) and permeabilization with 0.15% Triton-X100 in PBS. Following permeabilization, the cells were washed three times with PBS then blocked with Protein Block (Agilent/Dako, X090930-2). Primary antibodies [ADAR1 (Santa Cruz Biotechnology, catalog no. sc-73408, RRID:AB_2222767), Fibrillarin (Santa Cruz Biotechnology, catalog no. sc-25397, RRID:AB_640513), DDX54 (Novus, catalog no. NB100-60678, RRID:AB_921120), DDX17 (Thermo Fisher Scientific, catalog no. PA5-84585, RRID:AB_2791736), DHX9 (Bethyl, catalog no. A300-855A, RRID:AB_609442), ADAR1-p150 (Abcam, catalog no. ab126745, RRID:AB_11145661), dsRNA-J2 (Millipore, catalog no. MABE1134, RRID:AB_2819101) and secondary antibodies [Thermo Fisher Scientific, A21207 (RRID:AB_141637), A21203 (RRID:AB_2535789), A21202 (RRID:AB_141607), A21206 (RRID:AB_2535792)] were diluted in Antibody Diluent (Agilent/Dako, S302283-2). Antibody binding was performed in a humidity chamber for 1.5 hours for primaries and 30 minutes for secondaries. Between primary and secondary antibodies, and after secondary antibody binding the coverslips were washed in PBS. The coverslips were washed once in water before mounting on glass slides with Vectashield Antifade Mounting Media with DAPI (Vector Laboratories, H-1200-10). Fluorescence microscopy images were obtained with an Eclipse 90i microscope (Nikon) using a Plan Apochromatic 20x/NA 0.75 objective (Nikon) and a CoolSNAP ES2 monochrome digital camera cooled to 0°C (Photometrics), or a DM6 B microscope (Leica) with a 20x objective and a DFC90000 GT monochrome digital camera (Leica). Fluorescence images were captured with MetaMorph version 7.8.0.0 software (Molecular Devices, RRID:SCR_002368) or Leica Application Suite X (Leica, RRID:SCR_013673) and resized and formatted with Fiji (RRID:SCR_002285).

### Transfection of Poly(I:C)

The cell line indicated was transfected with high molecular weight poly(I:C; Invivogen) with Lipofectamine LTX (Invitrogen). A total of 3 µL of Lipofectamine LTX was used per microgram of poly(I:C). Sixteen to 24 hours after transfection, cells were harvested in RIPA with 1X HALT or the RNA lysis buffer from the Nucleospin RNA kit (Macherey-Nagel).

### RNA Purification and RNA Sequencing

RNA sequencing (RNA-seq) was performed for two replicates of ADAR1 and/or DHX9 knockdown in MCF-7 and SK-BR-3. RNA was purified using the Nucleospin RNA kit (Macherey-Nagel). Assessment of rRNA integrity and RNA-seq was performed by the Genome Technology Access Center at Washington University in St. Louis (St. Louis, MO). Total RNA integrity was determined using Agilent TapeStation 4200. Library preparation was performed with 500 ng to 1 µg of total RNA. Ribosomal RNA was removed by an RNase-H method using RiboErase kit (Kapa Biosystems). After rRNA depletion, the remaining RNA was then fragmented in reverse transcriptase buffer (Life Technologies) by heating to 94 degrees for 8 minutes. The RNA was reverse transcribed to yield cDNA using SuperScript III RT and random hexamers (Life Technologies) per manufacturer's instructions. A second strand reaction was performed with DNA Polymerase I and RNase H (Qiagen) to yield double-stranded cDNA (ds-cDNA). The cDNA was then blunted with T4 DNA Polymerase, Polynucleotide Kinase and Klenow DNA Polymerase (Qiagen). An A base was added to the 3′ ends with Klenow (3′–4′ exo-; Qiagen). The processed ds-cDNA was then ligated to Illumina sequencing adapters with T4 DNA Ligase (Qiagen). Ligated fragments were then amplified for 12–15 cycles using primers incorporating unique dual index tags with VeraSeq polymerase (Qiagen). Fragments were sequenced on an Illumina NovaSeq-6000 using paired end reads extending 150 bases.

### RNA-seq Analysis

The Illumina bcl2fastq (RRID:SCR_015058) software was used for base calling and demultiplexing, allowing for one mismatch in the indexing read. STAR version 2.7.9a1 (RRID:SCR_004463) was used for read alignment to RNA-seq to the Ensembl GRCh38.101 primary assembly. Gene counts were determined using Subread:featureCount version 2.0.32 (RRID:SCR_009803), only uniquely aligned unambiguous reads were counted. Differential gene expression was determined using DESeq2 (see Data Availability below for scripts). The experimental design for DESeq2 analysis included an interaction term between the shRNAs used for knockdown (“shrna1 + shrna2 + shrna1:shrna2”; where “shrna1” was either shSCR or shADAR and “shrna2” was either shSCR or shDHX9-3). Contrasts were used to assess differential expression after singular knockdown of either ADAR1 or DHX9. Fold changes were shrunken using the “apeglm” approach from DESeq2 (48). Gene set enrichment analysis was performed using “clusterProfiler” (RRID:SCR_016884; ref. 49). For analysis of transposable element expression, “TEcount” from TEtranscripts (RRID:SCR_023208; ref. 50) was used to determine family level counts for transposable elements using a GTF file containing transposable element information from RepeatMasker (http://www.repeatmasker.org, RRID:SCR_012954; see Data Availability section for more information about the GTF file).

### Foci Formation Assay

Five thousand cells were plated for each condition in a 10 cm culture dish. After 10 (BT-549, MDA-MB-MB231, HCC1806, MDA-MB-468, and SK-BR-3) to 20 (MCF-7) days the cells were washed briefly with 1x PBS prior to fixation in 100% methanol for 5 minutes. After drying, the cells were stained with 0.005% Crystal Violet solution containing 25% methanol (Sigma-Aldrich) prior to washing excess stain away with deionized water. The plates were scanned using an ImageScanner III (General Electric). Foci area was calculated using ImageJ (RRID:SCR_003070) or Fiji (RRID:SCR_002285).

### Quantitative PCR

Reverse transcription and qPCR were performed as described previously using iScript Supermix for cDNA synthesis and iTaq for qPCR (Bio-Rad; ref. 51). Fold change of RNA expression was determined using the ΔΔC_t_ method with normalization to PSMA5 and OAZ1. Primers are shown in Supplementary Table S1.

### Analysis of Cancer Cell Line Encyclopedia and The Cancer Genome Atlas Data

RNA-seq normalization and calculation of z-scores was performed as described previously (35). Molecular subtypes of breast cancer cell lines and The Cancer Genome Atlas (TCGA) samples were defined previously (52). Breast cancer survival analysis was performed using the R packages RTCGA and survminer (RRID:SCR_021094; ref. 53). The DHX9 expression level used for stratification in survival analysis was determined by the surv_cutpoint function of survminer.

### Data Availability Statement

Scripts used for analysis of MS, RNA-seq, and generation of all plots are available at (https://github.com/cottrellka/Cottrell-Ryu-et-al-2023), raw sequencing data and count files were deposited at the Gene Expression Omnibus under accession GSE224677. RNA-seq data for cancer cell lines (CCLE_expression_full.csv, CCLE_RNAseq_rsem_transcripts_tpm_20180929.txt) were obtained from the DepMap portal (https://depmap.org/portal/download/custom/, RRID:SCR_017655; ref. 54). RNAi-based dependency data for DHX9 (D2_combined_gene_dep_scores) were obtained from DepMap Portal (https://depmap.org/portal/download/custom/; ref. 55). RNA-seq data for TCGA breast cancer samples (illuminahiseq_rnaseqv2-RSEM_genes, illuminahiseq_rnaseqv2-RSEM_isoforms_normalized) and clinical data (Merge_Clinical) were obtained from the Broad Institute FireBrowse and are available at http://firebrowse.org/. The GTF file used for TEcount (GRCh38_Ensembl_rmsk_TE.gtf) is available at https://www.dropbox.com/sh/1ppg2e0fbc64bqw/AACUXf-TA1rnBIjvykMH2Lcia?dl=0.

## Results

### Proximity Labeling by APEX2 Reveals ADAR1-interacting Proteins

**FIGURE 1:**
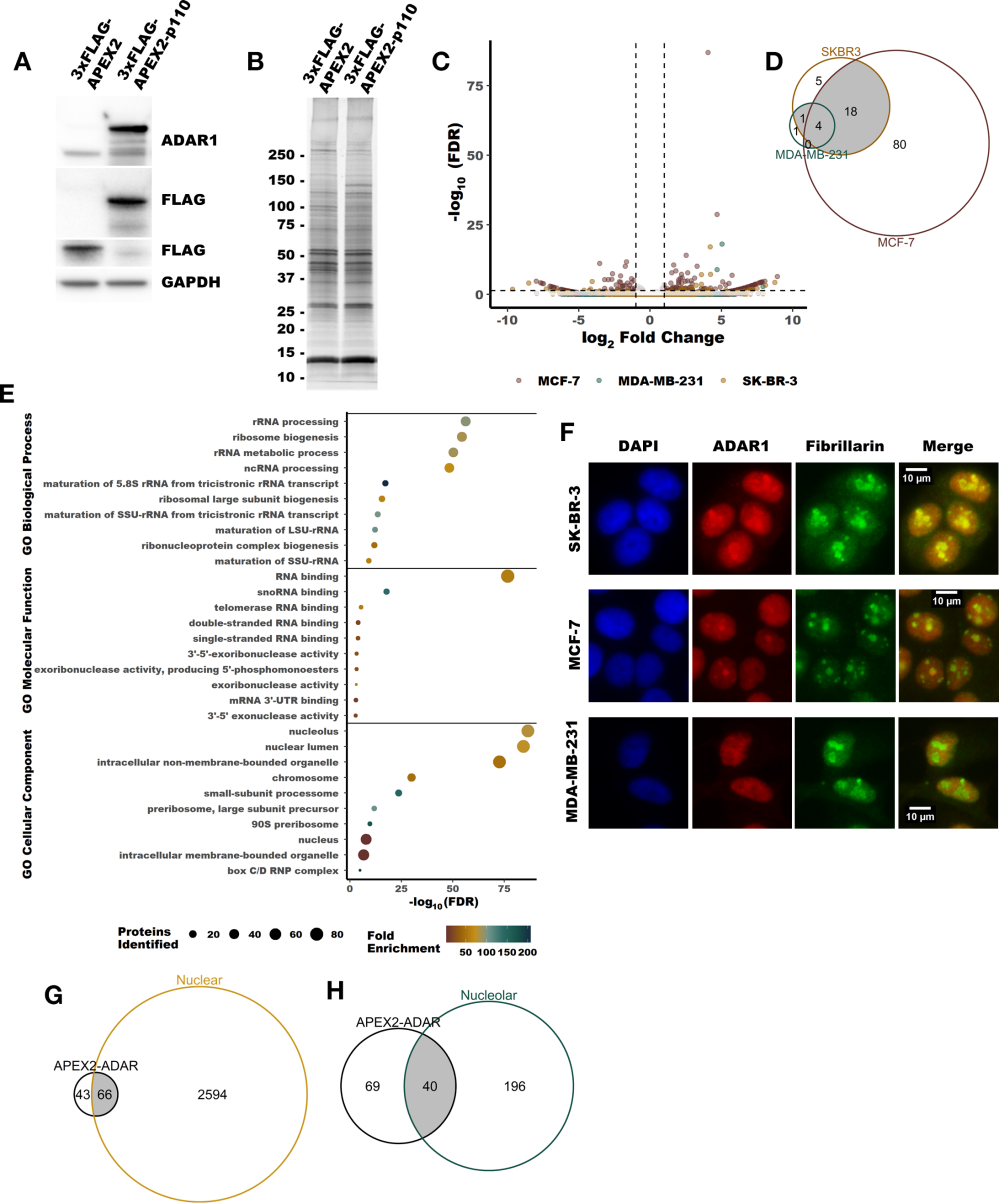
Identification of putative ADAR1-interacting proteins by APEX2 proximity labeling. **A,** Representative immunoblot showing expression of the constructs used for proximity labeling in MCF-7, MDA-MB-231 and SK-BR-3, MDA-MB-231 is shown. **B,** Representative fluorescently stained gel image showing proteins purified by streptavidin-biotin pulldown following proximity labeling in MCF-7. **C,** Volcano plot summarizing the proteins identified by MS following streptavidin pulldown subsequent to proximity labeling. Differential abundance of all proteins in each cell line can be found in Supplementary Table S2. **D,** Venn diagram showing overlap between enriched proteins from all three cell lines. The cutoff for enriched proteins was an FDR-adjusted *P* value of less than 0.05 and a log_2_ fold change of greater than 0.5. **E** and **F,** Venn diagrams showing overlap between the enriched proteins identified and those proteins found previously to localize to the nucleus or nucleolus in MCF-7 (57). **G,** GO terms found to be overrepresented in the list of the enriched proteins. Only the top 10 GO terms, by FDR, are shown for each category. All other significant GO terms are available in the Supplementary Tables S3 and S4. **H,** Representative indirect immunofluorescence micrographs showing localization of ADAR1, fibrillarin (a nucleolar marker) and DAPI.

To better understand the role of ADAR1-p110 in breast cancer, we turned to a proximity labeling approach using APEX2 to identify putative ADAR1-p110–interacting proteins. Proximity labeling by APEX2 allows for the identification of proteins within 20 nm of an APEX2 fusion protein via biotin-mediated pulldown (56). An APEX2-ADAR1-p110 fusion construct or APEX2 alone was expressed in MDA-MB-231, MCF-7, and SK-BR-3 (Fig. 1A). Following proximity labeling, biotinylated proteins were purified by streptavidin pulldown and subsequently identified by MS (Fig. 1B and C; Supplementary Fig. S1A; Supplementary Table S2). In total, we identified over 100 enriched proteins across the three cell lines (Fig. 1D). While the three cell lines used represent three subtypes of breast cancer (MDA-MB-231—triple-negative, SK-BR-3—HER2 positive, and MCF-7—estrogen receptor positive), there were several overlapping enriched proteins between the cell lines. For MDA-MB-231, five of the six enriched proteins overlapped with at least one other cell line. Similarly, 23 of 28 identified in SK-BR-3 overlapped with at least one other cell line. Overrepresentation analysis of gene ontology (GO) terms revealed that many of the proteins identified by proximity labeling have roles in multiple aspects of RNA metabolism and localize to the nucleus and nucleolus (Fig. 1E; Supplementary Tables S3–S5). This finding was consistent with the localization of ADAR1 (Fig. 1F, immunofluorescence for ADAR1 largely reflects localization of ADAR1-p110, which is the predominant isoform in these cell lines) and is supported by a comparison with proteins previously observed to localize to the nucleus and nucleolus within MCF-7 in the SubCellBarcode dataset (ref. 57; Fig. 1G and H).

### Validation of Protein Interactions Identified by Proximity Labeling

**FIGURE 2:**
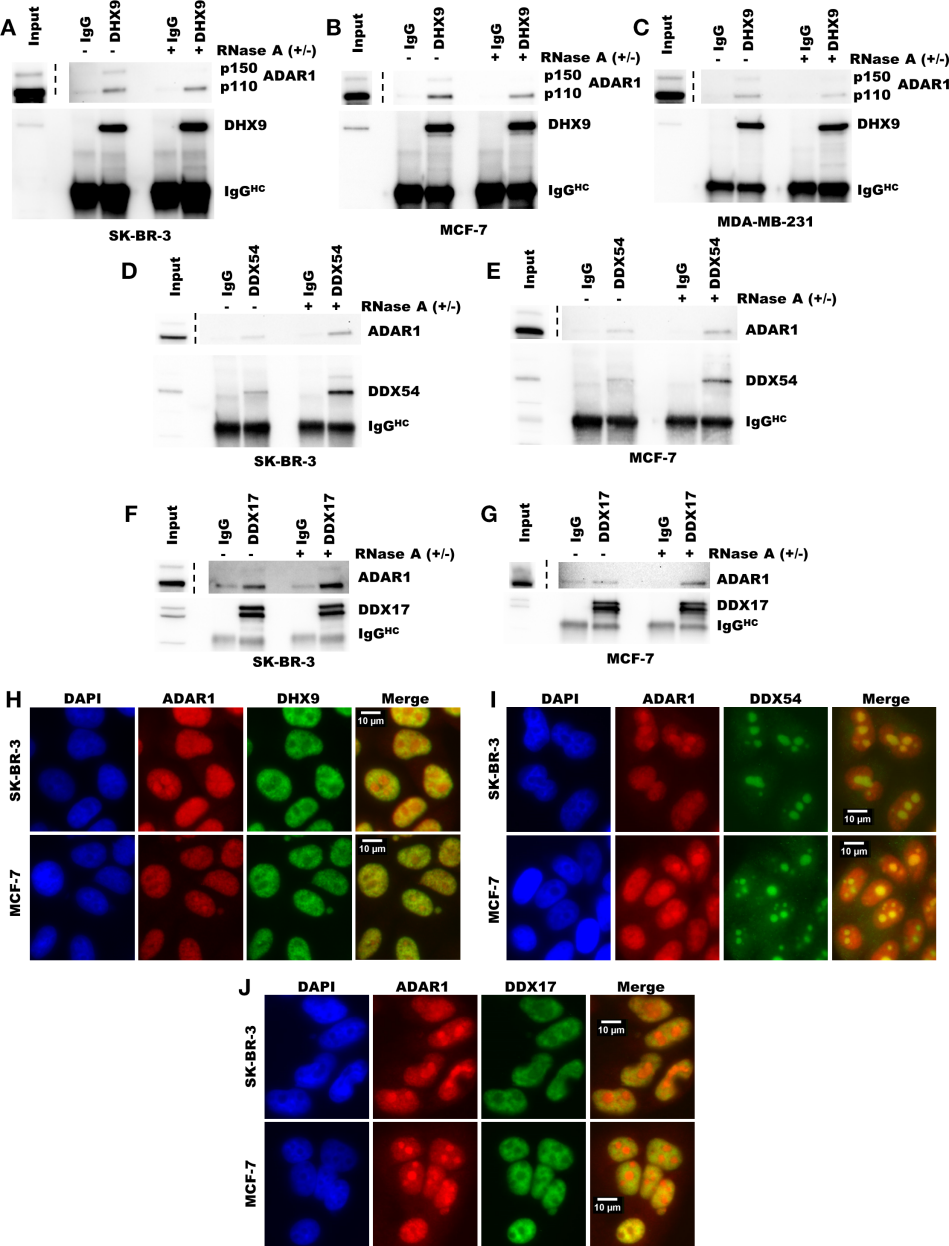
Validation of putative protein–protein interactions identified by proximity labeling. Immunoprecipitation of DHX9 (**A**–**C**), DDX54 (**D–E**), or DDX17 (**F–G**) followed by immunoblot for ADAR in breast cancer cell lines. Immunoblot of immunoprecipitation eluates and inputs from SK-BR-3 (A, D, and F), MCF-7 (B, E, and G), and MDA-MB-231 (C). Input represents 5% of the lysate used for immunoprecipitation. The IgG lanes represent immunoprecipitation eluates from pulldown with anti-rabbit IgG antibody. The lanes labeled DHX9, DDX54, and DDX17 indicate the eluates from immunoprecipitation with antibodies against those proteins, respectively. The IgG^HC^ label indicates the band corresponding to the IgG heavy chain from the antibody used for immunoprecipitation. Uncropped immunoblots for A–G can be found in Source Data Figures. Immunofluorescence for ADAR1 and DHX9 (**H**), DDX54 (**I**), or DDX17 (**J**) in SKBR3 or MCF-7.

Although proximity labeling by APEX2 is a powerful technique for identifying putative protein–protein interactions, it does not distinguish between interactions and close associations (56). To validate the proximity labeling findings and provide supporting evidence for direct protein–protein interactions, we performed co-immunoprecipitation followed by immunoblotting for five proteins identified by proximity labeling. ADAR1 could be immunoprecipitated by antibodies against the helicases DHX9, DDX17, and DDX54 (Fig. 2A–G). ADAR1 could also be immunoprecipitated with antibodies against XRN2 and PARP (Supplementary Fig. S1D and S1E). To assess whether these potential interactions depended on RNA, we treated the lysates with RNase A to degrade RNA prior to immunoprecipitation. Immunoprecipitation of ADAR1 by antibodies against DHX9, DDX17, and DDX54 was possible even in the presence of RNase A and improved in some cases (Fig. 2A–G). For DDX17 and DDX54, RNase A treatment did not change the co-immunoprecipitation results. While, DHX9 immunoprecipitated with both isoforms of ADAR1 in the absence of RNase A, degradation of RNA greatly reduced immunoprecipitation of ADAR1-p150 relative to ADAR1-p110 in MCF-7 and SK-BR-3 (Supplementary Fig. S1J). These findings suggest that DHX9, DDX17, and DDX54 directly interact with ADAR1-p110, and that in some cell lines a large portion of the DHX9 molecules interacting with ADAR1-p150 are doing so through an RNA bridge/scaffold. While RNase A has a preference for degradation of single-stranded RNA in the presence of salt, we observed that in the lysis buffer used for immunoprecipitation here, RNase A robustly degraded dsRNA as well (Supplementary Fig. S1H). RNase III treatment did not affect immunoprecipitation of ADAR1 isoforms along DHX9 (Supplementary Fig. S1F), it should however be noted that RNase III treatment did not degrade cellular RNA to the same extent as RNase A treatment (Supplementary Fig. S1G). The co-immunoprecipitation findings are also consistent with the localization of the proteins studied. Much like ADAR1, the helicases DHX9, DDX17, and DDX54 localized to the nucleus or nucleolus (Fig. 2H–J). Interestingly, in the cell lines used in this study, ADAR1-p150 is localized to the nucleus and cytosol (Supplementary Fig. S1C), consistent with previous reports of ADAR1-p150 shuttling between these two compartments (58, 59). Together, these findings validate the results of the proximity labeling described in Fig. 1 and provide evidence for direct interactions between ADAR1 and multiple helicases.

### DHX9 is Overexpressed in Breast Cancer

**FIGURE 3:**
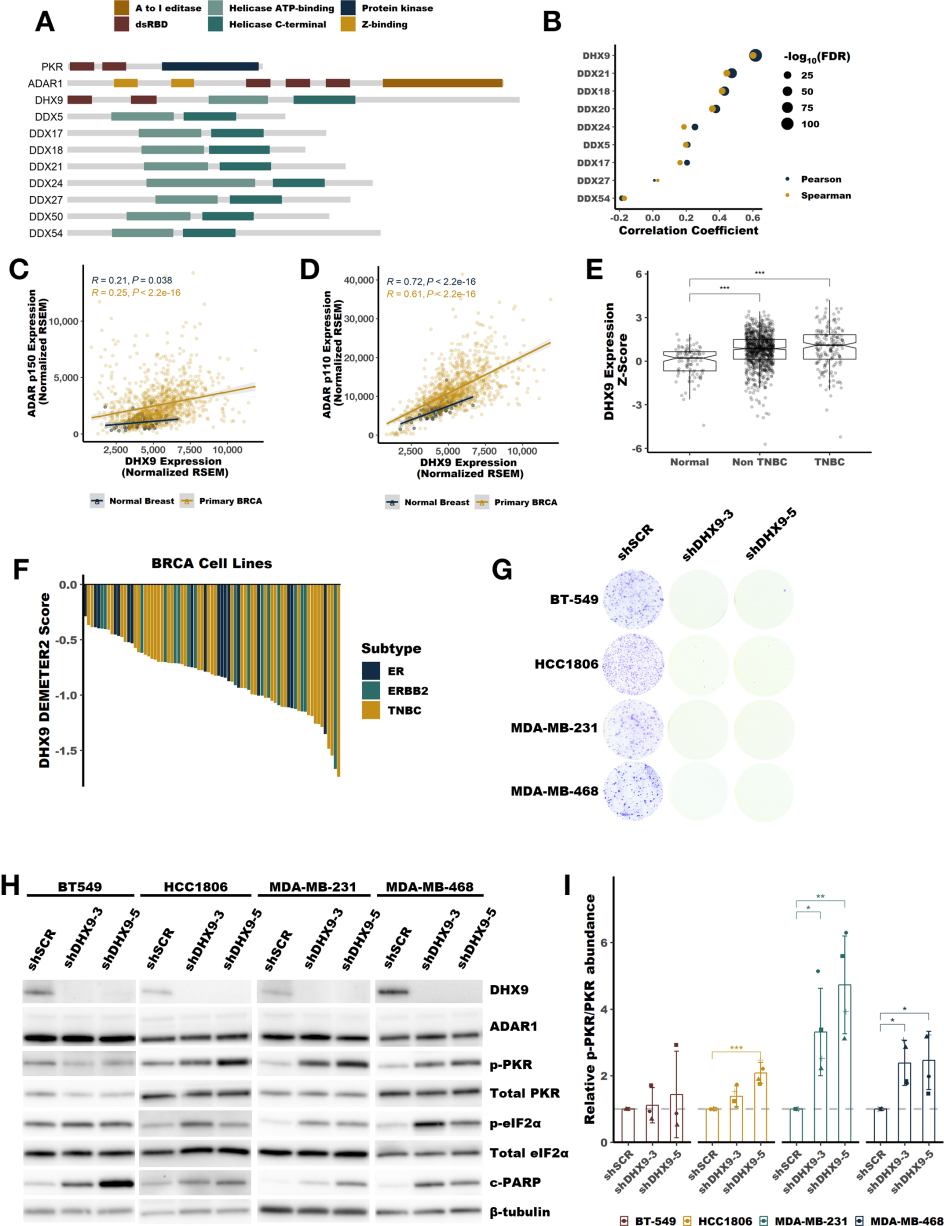
DHX9 is overexpressed in breast cancer and suppresses PKR activation. **A,** Schematic showing the domain structure of PKR, ADAR1, DHX9 and other helicases identified by proximity labeling in Fig. 1, dsRBD refers to the dsRNA binding domain. **B,** Pearson and Spearman correlation coefficients for the correlation between ADAR1 expression at the RNA level and the expression of each indicated helicase at the RNA level, data from breast tumors within TCGA. Scatterplots showing the correlation between ADAR1-p110 (**C**), or ADAR1-p150 (**D**), and DHX9 expression in normal breast or breast tumors. **E,** Expression of DHX9 at the RNA level in normal breast, non-TNBC or TNBC tumors. **F,** Waterfall plot showing DHX9 dependency of breast cancer cell lines using data from DepMap, ER = estrogen receptor–positive cell lines, ERRB2 = HER2-positive cell lines. **G,** Foci formation assay following knockdown of DHX9 with two different shRNAs in four TNBC cell lines. Cells were plated for foci formation 2 days after transduction and foci were stained after 10 days. **G,** Representative immunoblot following knockdown of DHX9 with two different shRNAs in four TNBC cell lines, same cells as used in G. Protein lysates were collected from cells 4 days after transduction with lentivirus encoding the shRNAs listed. Immunoblots for other replicates and uncropped blots can be found in Source Data Figures. **I,** Quantification of PKR phosphorylation as determined by the immunoblot in **H**. Quantification of protein expression for other proteins of interest can be found in Supplementary Fig. S4A–S4E. Bars represent the average of at least three replicates, error bars are ± SD. *, *P* < 0.05; **, *P* < 0.01; ***, *P* < 0.001. *P* values determined by Dunnett test.

Of the identified helicases, DHX9 was of particular interest for several reasons. First, DHX9 is the only DEAH or DEAD box helicase in humans that has a dsRBD. Nineteen human proteins contain dsRBDs as determined by sequence homology, including ADAR1 and PKR (Fig. 3A, https://prosite.expasy.org/rule/PRU00266). Second, analysis of publicly available RNA-seq data for human cell lines and tumors revealed that DHX9 expression closely correlates with ADAR1 expression (Fig. 3B–D; Supplementary Fig. S2B–S2F). The correlation is stronger between DHX9 and the transcript encoding ADAR1-p110, than between DHX9 and ADAR1-p150 (Fig. 3C and D; Supplementary Fig. S2D and S2E). Furthermore, the expression of DHX9 and ADAR1 correlates better than any other helicase identified by proximity labeling (Fig. 3B; Supplementary Fig. S2C). Consistent with this correlated expression, and much like ADAR1, DHX9 is highly expressed in breast cancer and is correlated with a poor prognosis (ref. 35; Fig. 3E; Supplementary Fig. S2G–S2I).

### DHX9 is Essential in TNBC Cell Lines and Suppresses PKR Activation

The similarities between ADAR1 and DHX9 led us to further study the role of DHX9 in breast cancer. Analysis of publicly available data from DepMap (https://depmap.org/portal/download/custom/) revealed that DHX9 is commonly essential in breast cancer cell lines (Fig. 3F). We validated this finding by knocking down DHX9 in four TNBC cell lines previously shown to be ADAR1 dependent (35). In all four lines, knockdown of DHX9 reduced foci formation (Fig. 3G). Analysis of cellular proliferation and viability over time following knockdown of DHX9 in MDA-MB-231 revealed an initial decrease in proliferation followed by reduced viability at later timepoints (Supplementary Fig. S3A and S3B). Apoptosis markers c-PARP [cleavage of PARP after Asp214 producing an 89 kDa fragment consistent with caspase activity (60, 61)], and Annexin V were elevated following knockdown of DHX9 (Fig. 3H; Supplementary Fig. S3C). Given the presence of the common dsRBD in DHX9 and PKR, and the role of DHX9 in regulating the abundance of dsRNA (62), we asked whether DHX9 could influence activation of PKR. To our surprise, we found that in three of the TNBC cell lines studied, knockdown of DHX9 caused activation of PKR (Fig. 3H and I), this finding and others described above were confirmed for MDA-MB-231 by CRISPR-Cas9 knockout of DHX9 (Supplementary Fig. S3D–S3F). Together, these findings show that DHX9 is essential in breast cancer cell lines and that in some ADAR1-dependent cell lines, DHX9 suppresses PKR activation.

### DHX9 and ADAR1 redundantly suppress PKR activation

**FIGURE 4:**
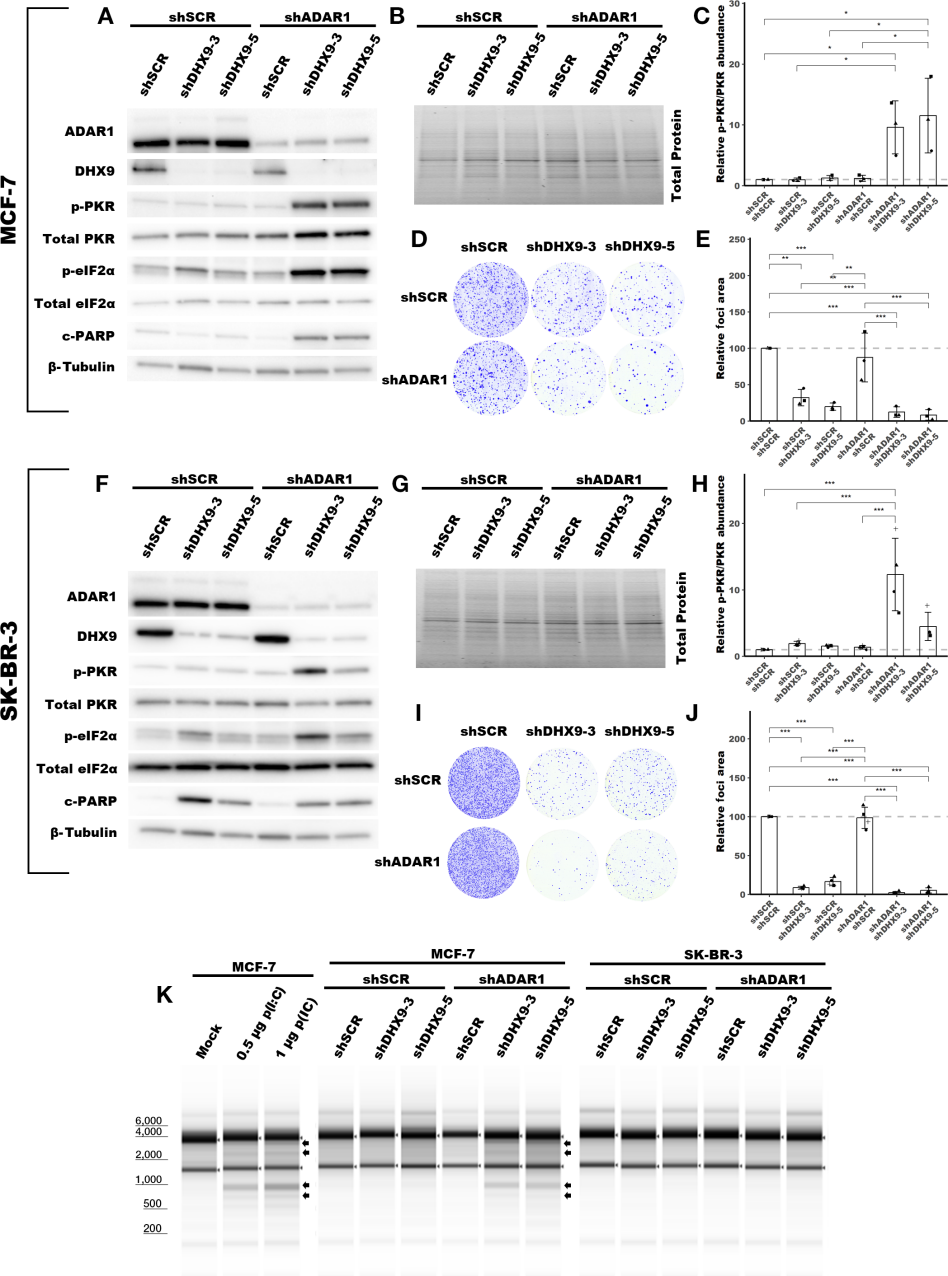
DHX9 and ADAR1 redundantly suppress dsRNA sensing in ADAR1-independent cell lines. Representative immunoblot showing the phenotype of ADAR1 and/or DHX9 knockdown in MCF-7 (**A**), or SK-BR-3 (**F**). Immunoblots for other replicates and uncropped blots can be found in Source Data Figures. Protein abundance from the immunoblot in **A** and **F** was normalized by total protein abundance by quantification of the Stain-Free gel in **B** and **G**, respectively. Fold change of PKR phosphorylation at Thr-446 in MCF-7 (**C**) or SK-BR-3 (**H**) as determined by the immunoblots in **A** or **F**, respectively. Quantification of protein expression for other proteins of interest can be found in Supplementary Fig. S5A–S5L. Protein lysates were collected from cells five (MCF-7) or four (SK-BR-3) days after transduction with lentivirus encoding the shRNAs listed. Representative foci formation phenotype of ADAR1 and/or DHX9 knockdown in MCF-7 (**D**) or SK-BR-3 (**I**), quantification of relative foci area is shown in **E** or **J**, respectively. Cells were plated for foci formation 2 days after transduction and foci were stained when visible, 21 (MCF-7) or 10 (SK-BR-3) days later. **K,** Analysis of rRNA integrity upon knockdown of ADAR1 and/or DHX9 in MCF-7 or SK-BR-3. RNA was isolated from cells at the same time protein lysates were collected. In addition, **K** shows the effect of poly(I:C) [p(I:C)] transfection on rRNA integrity in MCF-7. Arrows indicate canonical RNase L cleavage products (64). Bars represent the average of at least three replicates, error bars are ± SD. *, *P* < 0.05; **, *P* < 0.01; ***, *P* < 0.001. *P* values determined by one-way ANOVA with *post hoc* Tukey. Comparisons between the two different shRNAs targeting DHX9 (shDHX9-3 and shDHX9-5) were not included for clarity.

The experiments above were performed in ADAR1-dependent TNBC cell lines—cell lines that activate PKR following ADAR1 knockdown (35). We were curious whether DHX9 knockdown would also cause PKR activation in ADAR1-independent cell lines—cell lines that do not activate PKR following ADAR1 knockdown. Using shRNAs, we knocked down DHX9 in two ADAR1-independent breast cancer lines, MCF-7 and SK-BR-3 (Fig. 4A–B and F–G; Supplementary Fig. S5A–S5B and S5G–S5H). Unlike in ADAR1-dependent cell lines, knockdown of DHX9 did not cause activation of PKR in ADAR1-independent cell lines (Fig. 4A, C, F, and H). Next, we asked whether combined knockdown of ADAR1 and DHX9 in these cell lines would lead to activation of PKR. As we had previously observed, knockdown of ADAR1 in SK-BR-3 and MCF-7 did not cause PKR activation (35). However, combined knockdown of DHX9 and ADAR1 caused robust activation of PKR in both cell lines (Fig. 4A, C, F, and H). Consistent with PKR activation, we observed increased phosphorylation of the PKR substrate eIF2ɑ following combined knockdown of ADAR1 and DHX9 (Fig. 4A and F; Supplementary Fig. S5C and S5I). Like in ADAR1-dependent TNBC cell lines, knockdown of DHX9 caused reduced foci formation of MCF-7 and SK-BR-3 (Fig. 4D, E, I, and J), likely through caspase-dependent apoptosis, as indicated by elevated cleaved PARP (Fig. 4A and F; Supplementary Fig. S5E and S5K). Although PARP cleavage was increased upon combined knockdown of DHX9 and ADAR1, this did not statistically reduce foci formation of the cells compared with single knockdown of ADAR1 and DHX9 as measured by foci formation. Together, these results reveal that ADAR1 and DHX9 redundantly suppress PKR activation in ADAR1-independent breast cancer cell lines.

### DHX9 and ADAR1 Redundantly Suppress Multiple Innate Immunity Pathways

Next, we wanted to evaluate the activation of other dsRNA sensing pathways in ADAR1-independent cell lines after the combined knockdown of ADAR1 and DHX9. To assess whether the IFN-I pathway, or other pathways, is activated after combined knockdown of ADAR1 and DHX9 we turned to analysis of differential gene expression by RNA-seq. In the process of preparing RNA for sequencing, we were surprised to find specific degradation of rRNA in MCF-7 cells following combined knockdown of ADAR1 and DHX9 (Fig. 4K). Single knockdown of either DHX9 or ADAR1 did not cause rRNA degradation. The observed rRNA degradation in the combined knockdown cells is consistent with the degradation products caused by RNase L, as indicated in Fig. 4K (63, 64). Transfection with poly(I:C), which activates the IFN-I pathway and RNase L (65), created an identical band pattern to that of combined knockdown of DHX9 and ADAR1, indicating that the degradation of rRNA observed in these cells is likely caused by RNase L activity (Fig. 4K). We performed the same experiment with the ADAR1-dependent TNBC cell lines described above. In these cells, we did not see rRNA degradation consistent with RNase L activity after the knockdown of DHX9 alone (Supplementary Fig. S4H). RNase L is activated by 2′,5′-oligoadenylate generated by the OAS proteins (OAS1, OAS2, and OAS3; ref. 24). From our RNA-seq data, described below, we observed increased expression of the OASs in MCF-7 after double knockdown of DHX9 and ADAR1 (Supplementary Table S8). In fact, a GO term associated with OAS activity was upregulated in MCF-7 after combined knockdown of ADAR1 and DHX9 (Supplementary Table S14).

**FIGURE 5:**
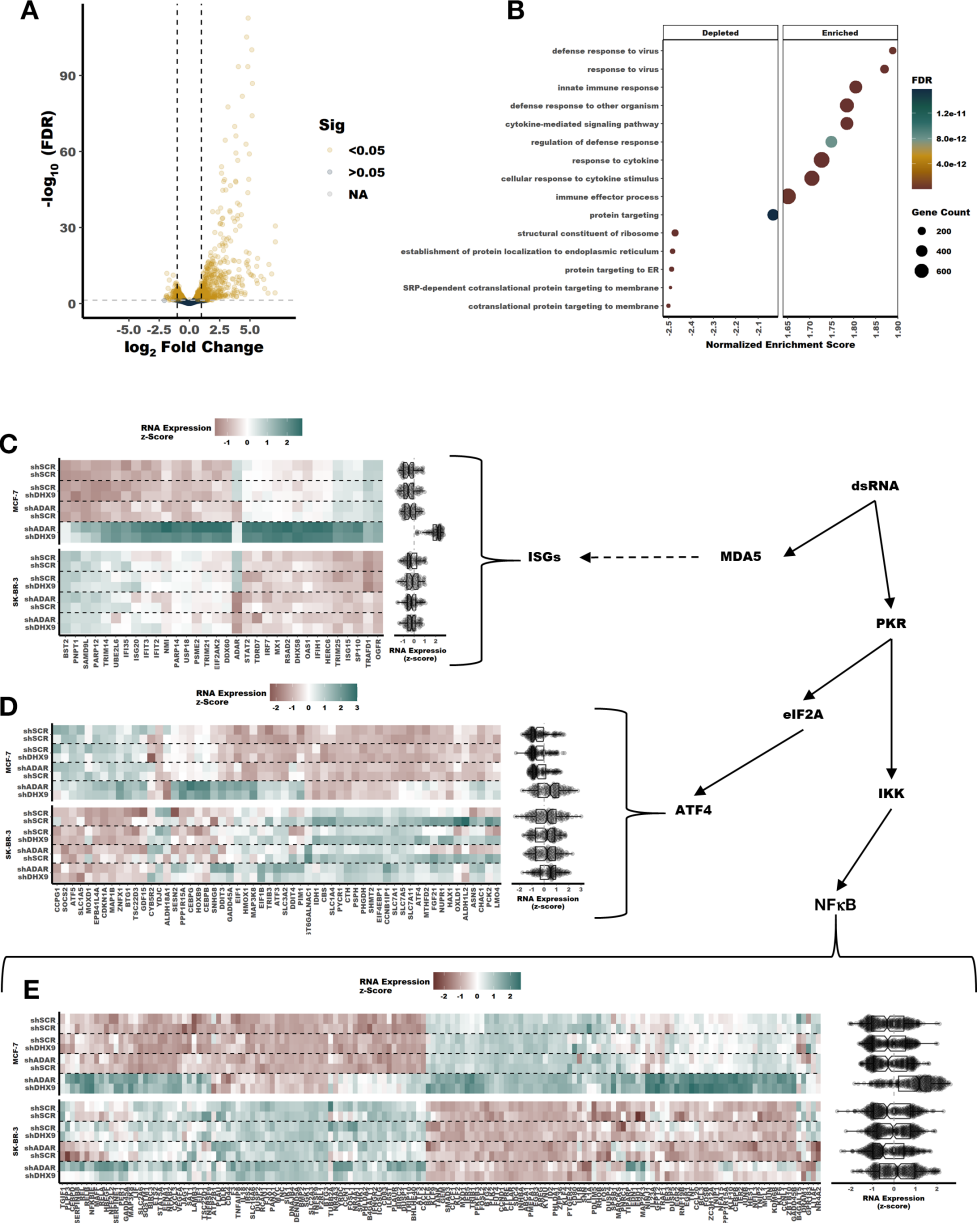
Induction of a viral mimicry phenotype upon knockdown of DHX9 and ADAR1 in MCF-7. **A,** Volcano plot showing changes in RNA expression upon knockdown of DHX9 and ADAR1 in MCF-7, a volcano plot for SK-BR-3 is in Supplementary Fig. S6F. RNA was isolated from cells 5 days after transduction with the shRNAs listed. Fold change of RNA expression shown in A was determined using an interaction term between ADAR1 and DHX9 knockdown, volcano plots for fold change of RNA expression for single knockdown of ADAR1 or DHX9 is in Supplementary Fig. S6A–S6B and S6D–S6E. **B,** GO terms identified by gene set enrichment analysis of the RNA-seq data in A. **C–E,** Heat maps and summary box plots showing RNA expression changes in MCF-7 and SK-BR-3 upon knockdown of ADAR1 and/or DHX9. **C** shows RNA expression for core ISGs (22, 35), **D** shows ATF4 targets, and **E** shows NFκB targets with ISGs removed. Genes are clustered by RNA expression across all four samples. Clustering was performed for each gene set (ISGs, ATF4 targets, or NFκB targets) independently. For more information, see GitHub repository link in Data and Code Availability.

RNA-seq revealed that many more RNAs were differentially expressed after combined knockdown of DHX9 and ADAR1, compared with single knockdown of ADAR1 or DHX9 (Fig. 5A; Supplementary Fig. S6A–S6F). Analysis of differential gene expression by gene set enrichment after combined knockdown of ADAR1 and DHX9 in MCF-7 revealed activation of multiple pathways involved in the innate response to viral infection and repression of several pathways involved in translation (Fig. 5B; Supplementary Table S14). An enrichment map showed that the activated pathways associated with innate immunity formed one cluster and the depressed pathways formed a separate cluster (Supplementary Fig. S6G). Of the upregulated pathways, several were associated with activation of IFN signaling (Fig. 5B; Supplementary Table S14). Analysis of core ISG expression revealed significant upregulation of ISGs in MCF-7 after combined knockdown of ADAR1 and DHX9 (Fig. 5C; Supplementary Fig. S6C). On the contrary, knockdown of DHX9 or ADAR1 alone did not increase ISG expression. Consistent with activation of PKR, we also observed increased expression of ATF4 targets (Fig. 5D) and NFκB targets (Fig. 5E). Interestingly, we did not observe activation of any of these pathways or rRNA degradation consistent with RNase L activity in SK-BR-3 following combined ADAR1 and DHX9 knockdown. It is not clear from these data what causes this difference between SK-BR-3 and MCF-7. SK-BR-3 expresses MDA-5, MAVS, and PKR to a similar level as MCF-7 (Supplementary Fig. S7A). Furthermore, both cell lines respond to transfection of p(I:C) by activation of PKR and the IFN-I pathway as indicated by increased expression of PKR and MDA5 (Supplementary Fig. S7B).

The finding that combined knockdown of ADAR1 and DHX9 did not induce ISG expression in SK-BR-3 is consistent with our findings in TNBC cell lines. While knockdown of DHX9 alone caused activation of PKR in several TNBC cell lines, we observed no activation of the IFN-I pathway, as indicated by no change in ISG15 expression (Supplementary Fig. S4F and S4G). ISG15 was found to be highly upregulated at the RNA and protein level in MCF-7 after double knockdown of ADAR1 and DHX9 (Supplementary Fig. S5F and S5M; Supplementary Table S8). Like ISG expression overall, ISG15 expression in SK-BR-3 was not changed by knockdown of DHX9 and ADAR1 (Supplementary Fig. S5L and S5N).

Given the previously described role of DHX9 in the control of Alu containing RNAs (62), we next sought to assess whether increased expression of transposable elements, especially Alus, could explain the activation of PKR or the IFN pathway upon combined knockdown of DHX9 and ADAR1. Analysis of our RNA-seq data revealed that transposable element expression was generally unchanged upon either single knockdown of ADAR1 or DHX9, or combined knockdown of both proteins (Supplementary Fig. S7C–S7H).

### The dsRBDs of DHX9 are Sufficient to Suppress PKR Activation in the Absence of ADAR1

**FIGURE 6:**
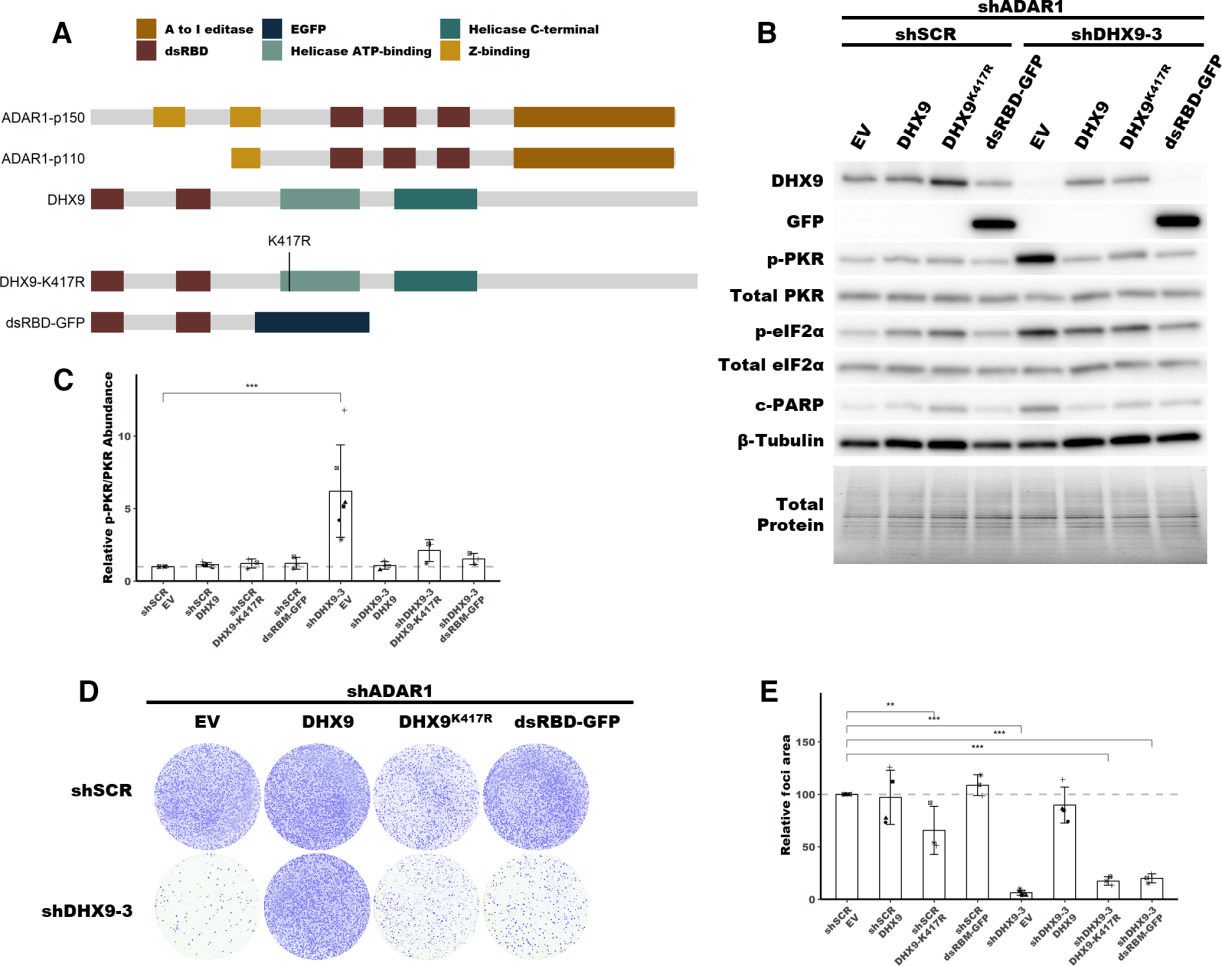
Rescue of PKR activation by DHX9 mutants in SK-BR-3. **A,** Schematic showing the domain structure of ADAR1 isoforms, DHX9 and mutants of DHX9, dsRBD refers to the dsRNA binding domain. **B,** Representative immunoblot showing the phenotype of ADAR1 and DHX9 knockdown with DHX9, DHX9^K417R^ or dsRBD-EGFP overexpression in SK-BR-3. Immunoblots for other replicates and uncropped blots can be found in Source Data Figures. **C,** Fold change of PKR phosphorylation at Thr-446 upon ADAR1 and DHX9 knockdown with DHX9, DHX9^K417R^ or dsRBD-EGFP overexpression in SK-BR-3, quantified from immunoblots in B. Quantification of protein expression for other proteins of interest can be found in Supplementary Fig. S8B–S8F. **D,** Representative foci formation phenotype of ADAR1 and DHX9 knockdown with DHX9, DHX9^K417R^ or dsRBD-EGFP overexpression in SK-BR-3. Quantification of relative foci area is shown in **E**. Timepoints for collecting protein lysates and foci formation are the same as described in Fig. 4 for SK-BR-3. Bars represent the average of at least three replicates, error bars are ± SD. *, *P* < 0.05; **, *P* < 0.01; ***, *P* < 0.001. *P* values determined by Dunnett test.

**FIGURE 7:**
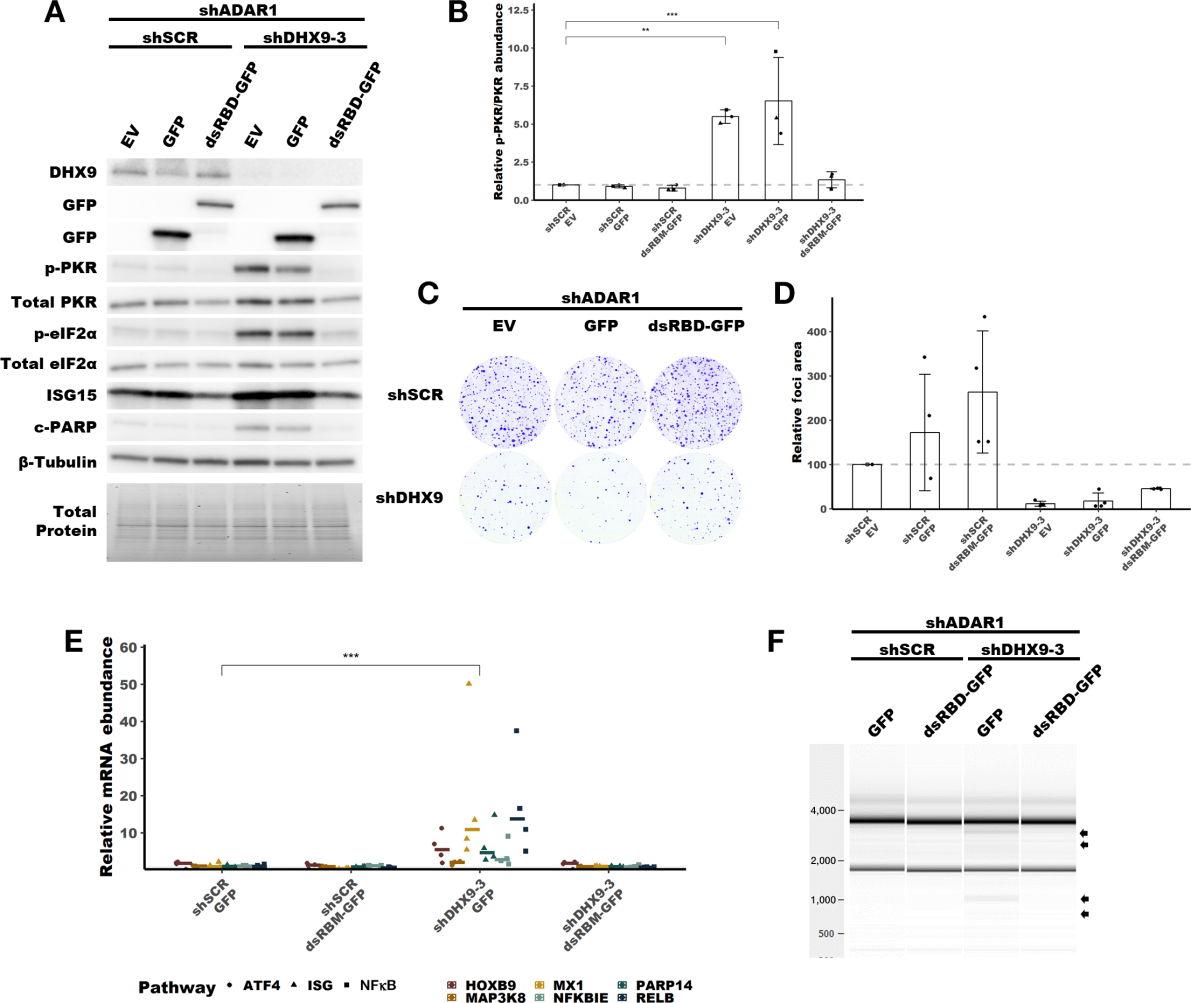
Rescue of PKR activation by DHX9 mutants in MCF-7. **A,** Representative immunoblot showing the phenotype of ADAR1 and DHX9 knockdown with DHX9, DHX9^K417R^ or dsRBD-EGFP overexpression in MCF-7. Immunoblots for other replicates and uncropped blots can be found in Source Data Figures. **B,** Fold change of PKR phosphorylation at Thr-446 upon ADAR1 and DHX9 knockdown with DHX9, DHX9^K417R^ or dsRBD-EGFP overexpression in MCF-7, quantified from immunoblots in A. Quantification of protein expression for other proteins of interest can be found in Supplementary Fig. S9B–S9G. **C,** Representative foci formation phenotype of ADAR1 and DHX9 knockdown with DHX9, DHX9^K417R^ or dsRBD-EGFP overexpression in MCF-7. Quantification of relative foci area is shown in **D**. **E,** RNA expression of representative genes from upregulated pathways in Fig. 5 as determined by qRT-PCR for the MCF-7 rescue experiment. Statistical analysis for each pathway is shown in Supplementary Fig. S9H–S9J. **F,** Analysis of rRNA integrity upon knockdown of ADAR1 and DHX9 in MCF-7 with overexpression of EGFP or dsRBD-EGFP. Arrows indicate canonical RNase L cleavage products (64). Timepoints for collecting protein lysates, isolating RNA, and foci formation are the same as described in Fig. 4 for MCF-7. Bars represent the average of at least three replicates, error bars are ± SD. *, *P* < 0.05; **, *P* < 0.01; ***, *P* < 0.001. *P* values determined by Dunnett test.

Having shown through knockdown studies that ADAR1 and DHX9 function redundantly to suppress dsRNA sensing, we next wanted to assess which functions of DHX9 and which isoforms of ADAR1 are important for this role. To determine which functions of DHX9 are sufficient to suppress PKR activation, we performed a rescue experiment with wild-type DHX9, a helicase deficient mutant DHX9, and a truncated DHX9 that possess the N-terminal dsRBDs fused to EGFP (Fig. 6A; Supplementary Fig. S8A). Overexpression of wild-type DHX9 in SK-BR-3 rescued the PKR activation phenotype caused by double knockdown of ADAR1 and DHX9, confirming that the observed phenotypes are not an off-target effect of the shRNAs used for knockdown (Fig. 6B–E). Interestingly, the DHX9^K417R^ mutant, which lacks helicase activity due to its inability to bind ATP (66), was also capable of suppressing PKR activation. The same was true for a construct which contained the dsRBDs of DHX9 fused to EGFP (dsRBD-EGFP). Rescue experiments in MCF-7 revealed that expression of the DHX9 dsRBD-EGFP fusion protein was sufficient to suppress not only activation of PKR, but also the RNA expression pathways described in Fig. 5 and rRNA degradation consistent with RNase L activity, but like in SK-BR-3 did not rescue the foci formation phenotype (Fig. 7A–F; Supplementary Fig. S9). These findings indicate that the DHX9 dsRBDs are likely sufficient to suppress activation of PKR and the IFN-I pathway in the absence of ADAR1 and DHX9. However, only wild-type DHX9 could rescue the reduced foci formation observed after DHX9 knockdown (Figs. 6D–E and 7C–D). This finding indicates that the PKR activation phenotype and the reduced proliferation phenotype are uncoupled.

Consistent with the observation that the DHX9 dsRBDs are sufficient to suppress PKR activation, knockdown of DDX17, which lacks dsRBDs, did not cause substantial PKR activation in SK-BR-3 (Supplementary Fig. S10A). Unlike DHX9, knockdown of DDX17 had no effect on cell proliferation as measured by the foci formation assay (Supplementary Fig. S10B and S10C). While combined knockdown of DHX9 and ADAR1 in SK-BR-3 caused a 5- to 10-fold increase in PKR phosphorylation, the combined knockdown of DDX17 and ADAR1 caused only a modest 1.5-fold increase in PKR phosphorylation (Supplementary Fig. S10A and S10D). This finding underscores the novel role of the helicase DHX9 and its dsRBD, a unique domain among this large family of RNA helicases.

### The p110 and p150 Isoforms of ADAR1 Suppress PKR Activation in the Absence of DHX9

**FIGURE 8:**
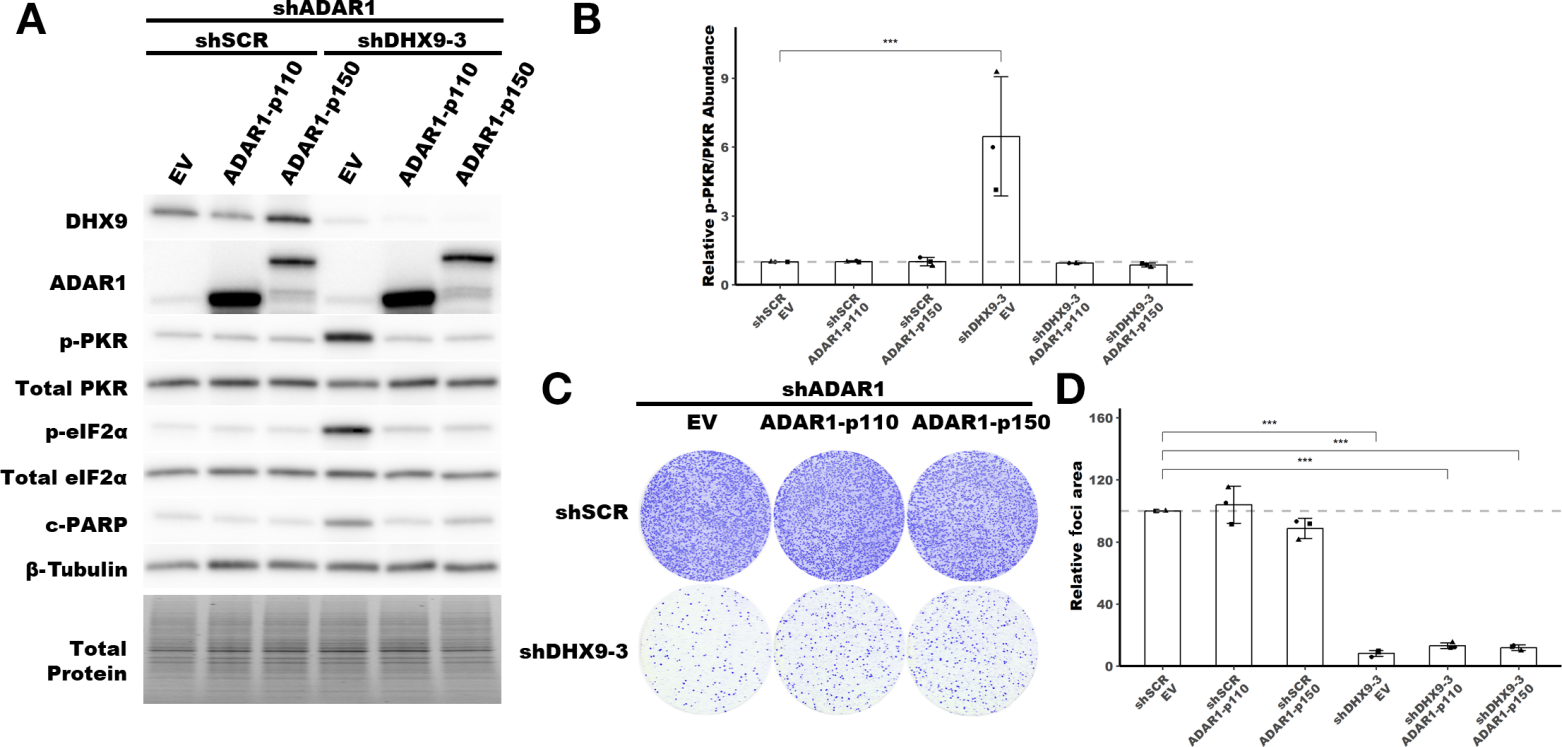
Rescue of PKR activation by ADAR1-p110 and ADAR-p150. **A,** Representative immunoblot showing the phenotype of ADAR1 and DHX9 knockdown with ADAR1-p110 or ADAR1-p150 overexpression in SK-BR-3. Immunoblots for other replicates and uncropped blots can be found in Source Data Figures. **B,** Fold change of PKR phosphorylation at Thr-446 upon ADAR1 and DHX9 knockdown with ADAR1 isoform overexpression, quantified from immunoblots in A. **C,** Representative foci formation phenotype of ADAR1 and DHX9 knockdown ADAR1 isoform overexpression. Quantification of relative foci area is shown in **D**. Quantification of protein expression for other proteins of interest can be found in Supplementary Fig. S11A–S11F. Timepoints for collecting protein lysates and foci formation are the same as described in Fig. 4 for SK-BR-3. Bars represent the average of at least three replicates, error bars are ± SD. ***, *P* < 0.001. *P* values determined by Dunnett test.

Next, we turned to ADAR1, and asked which isoform of ADAR1 is sufficient to suppress PKR activation in the absence of DHX9. We used the same approach as above, a rescue experiment with overexpression of ADAR1-p110 or ADAR1-p150. Interestingly, we found that both ADAR1 isoforms were sufficient to suppress PKR activation upon loss of DHX9 (Fig. 8A and B; Supplementary Fig. S11). However, overexpression of neither ADAR1 isoform was able to rescue the foci formation phenotype, again indicating that the PKR activation and cell proliferation phenotypes are uncoupled (Fig. 8C and D).

## Discussion

In recent years, ADAR1 has become an important therapeutic target for breast and other cancers. It is clear from the literature that depletion of ADAR1 in ADAR1-dependent cell lines leads to activation of dsRNA sensors and innate immunity programs that lead to cell death (22, 34, 35). Yet unclear is what distinguishes ADAR1-dependent cell lines from ADAR1-independent cell lines—those that are insensitive to ADAR1 depletion. Elevated ISG expression has been proposed as a potential prerequisite for ADAR1-dependency, but some ADAR1-independent cell lines exhibit elevated ISG expression (22, 35). As such, more information is needed to identify the factors that establish ADAR1-dependency or ADAR1-independency. To begin to fill in some of the knowledge gaps surrounding ADAR1, we utilized proximity labeling to identify putative ADAR1-interacting proteins, specifically focusing on the less studied ADAR1-p110 isoform.

Of the proteins identified by proximity labeling, the DEAH box helicase DHX9 was of particular interest. Like ADAR1 and PKR, DHX9 possesses dsRBDs, a singularly unique trait among the DEAD/DEAH box RNA helicase family. Like many RNA helicases, DHX9 is important in numerous cellular processes, ranging from mRNA processing to resolution of R-Loops (67, 68), and has been shown to promote antiviral immunity by activation of the IFN-I, though those studies were not performed using cancer cell lines (69, 70). DHX9 expression is strongly correlated with ADAR1-p110 expression in breast cancer. Both genes are located on the q-arm of chromosome 1, though they are separated by 28 Mb and are thus unlikely to be physically coregulated. Consistent with other reports in the literature, we show here that ADAR1 and DHX9 likely interact directly (40). While future experiments are needed to verify that ADAR1 interacts directly with DHX9, the data described here support an RNA-independent interaction between ADAR1 and DHX9. However, the importance of the ADAR1–DHX9 interaction is unclear from this work. Given that knockdown of either DHX9 or ADAR1 alone can induce PKR activation in ADAR-dependent cell lines, and that knockdown of both proteins is required to do the same in ADAR-independent cell lines, it seems unlikely that the interaction between ADAR1 and DHX9 is required for suppression of dsRNA sensing. That of course does not preclude other functions for a DHX9–ADAR1 complex, possibly in resolution of R-loops as both proteins have been implicated in R-loop homeostasis (29, 67). More studies are needed to structurally assess the interaction and directly perturb the interaction to understand what function it may have.

Here we report that in addition to being an essential gene in breast cancer, DHX9 suppresses dsRNA sensing (see Supplementary Fig. S13 for a hypothetical mechanism). In ADAR1-dependent cell lines, knockdown of DHX9 alone—much like knockdown of ADAR1 alone—caused activation of the dsRNA sensor PKR (35). Like ADAR1 knockdown, DHX9 knockdown had no effect on PKR activation in ADAR1-independent cell lines. However, combined knockdown of DHX9 and ADAR1 caused robust activation of PKR in those cells. This finding indicates that ADAR1 and DHX9 function redundantly to suppress PKR activation in ADAR1-independent cell lines and provides an explanation for why PKR is not activated in ADAR1-independent cells upon ADAR1 knockdown. In addition to suppression of PKR activation, we also observed that DHX9 and ADAR1 redundantly suppress activation of several other dsRNA sensing pathways in MCF-7. Knockdown of both proteins caused activation of IFN-I signaling, likely via MDA5 activation, as previously shown for ADAR1 (13, 15). We also observed OAS expression and rRNA degradation consistent with RNase L activation, and increased expression of the ATF4 and NFκB targets, likely downstream of PKR activation (71–73). Taken together, combined knockdown of DHX9 and ADAR1 in MCF-7 creates a viral mimicry phenotype, where multiple innate immunity pathways against RNA viruses have been activated. In contrast to MCF-7, we only observed activation of PKR in other cell lines after either DHX9 knockdown alone or combined knockdown with ADAR1. Transfection of the same cell lines with poly(I:C) revealed that each cell line could activate PKR, the IFN-I pathway and cause rRNA degradation consistent with RNase L activity (Supplementary Fig. S7A and S7B), which suggests that other variables are preventing activation of those pathways upon loss of DHX9 and ADAR1 in unresponsive cell lines. Given that PKR can be activated by much shorter dsRNAs [>30 bp (74)] than MDA5 [>500 bp (75)], it is possible that a different set of RNAs are responsible for activation of each pathway. RNA-modifying enzymes like APOPBEC3B and METTL3 have been shown to influence activation of dsRNA sensors like PKR in both an activating and suppressing manner, respectively (76, 77). The expression of those proteins, other dsRBPs, or dsRNA sensors like ZBP1 may influence how sensitive a cell line is to loss of DHX9 or ADAR1. Further studies are needed to identify other proteins that function to suppress dsRNA sensing, and to identify the dsRNAs that are activating the various pathways.

Rescue experiments revealed that the helicase activity of DHX9 was dispensable for suppression of PKR activation, and that the N-terminal dsRBDs of DHX9 were sufficient to suppress PKR activation in the absence of ADAR1 in ADAR1-independent cell lines. These data are consistent with a model in which DHX9 competes with PKR for dsRNA binding. This competition could come in the form of DHX9 binding directly to PKR and preventing its binding to dsRNA, or DHX9 binding dsRNA and preventing PKR binding. While DHX9 and PKR interact, and in fact DHX9 is phosphorylated by PKR (78), it is our opinion that a protein–protein interaction model is less likely considering that DHX9 is nuclear localized whereas PKR is largely localized to the cytoplasm (68, 79–81). Conversely, DHX9 may compete with PKR for binding of dsRNA and do so by sequestering some dsRNAs within the nucleus. Sequestration of some dsRNAs within the nucleus is an important means of preventing dsRNA sensors activation. During mitosis, nuclear dsRNAs diffuse into the cytosol and activate PKR (82). Interestingly, we did not observe a global shift in dsRNA localization following knockdown of DHX9 and ADAR1 in MCF-7 (Supplementary Fig. S12E and S12F). This finding may suggest that only a small subset of dsRNAs is responsible for activation of dsRNA sensors following loss of ADAR1 and DHX9 in those cells. This hypothesis is consistent with previous studies of ADAR1 depletion in which it was observed that only 2% of A-to-I edits are required to prevent MDA5 activation (83). The hypothesis that only a subset of endogenous dsRNAs is responsible for activation PKR or MDA5 following loss of ADAR1 and/or DHX9 could also explain the varied results seen here across different cell lines. Some cell lines may be more sensitive to loss of ADAR1 and/or DHX9 based on the expression of specific endogenous dsRNAs.

Previously, the ADAR1 p150 isoform, and not the p110 isoform, was shown to be responsible for suppression of PKR activation (19, 34). Through rescue experiments, we show here that both isoforms are sufficient to suppress PKR activation in the absence of DHX9 in ADAR1-independent cell lines. It is important to note that the role of ADAR1-p110 in suppressing PKR activation has likely been missed previously due to the expression of DHX9. The finding that ADAR1-p110 can suppress PKR activation in the absence DHX9 highlights a redundant role of these nuclear dsRNA binding proteins. An article that was published during the preparation of this article showed that the dsRBDs of ADAR1, ADAR2, and STAU1 were sufficient to suppress PKR activation (84). In addition, we and others have previously shown that the phenotype of ADAR1 depletion in ADAR1-dependent cells could at least be partially rescued by overexpression of an editing deficient ADAR1 (34, 35). On the basis of these findings and our rescue experiment with the DHX9 dsRBDs, it is likely that ADAR1-p150 and ADAR1-p110 suppress dsRNA sensing by competing with PKR for dsRNA binding. For the ADAR1-p150 isoform, this competition with PKR for dsRNA binding is likely to be direct due to the cytoplasmic localization of ADAR1-p150 and PKR. However, for the nuclear localized ADAR1-p110 (Supplementary Fig. S12D), we propose that like DHX9, ADAR1-p110 may function to sequester PKR activating dsRNAs in the nucleus. Interestingly, the DHX9-dsRBD-EGFP fusion protein, which we showed was capable of suppressing PKR activation, localizes to the cytoplasm (Supplementary Fig. S12C). Together these findings suggest that there may be multiple mechanisms to prevent PKR activation—direct competition, like ADAR1-p150; and sequestration of dsRNAs, as may be the case for ADAR1-p110 and DHX9.

Our rescue experiments revealed that while the helicase activity of DHX9 was dispensable for suppression of dsRNA sensing, it was required for cell viability. Given that DHX9 has been shown to have an important role in many cellular processes, ranging from processing of mRNAs to resolution of R-Loops, we suspect the reduced viability associated with loss of DHX9 helicase activity is related to one or more of these additional DHX9 roles (67, 68) During revisions of this article, a report was published showing that depletion of DHX9 in lung cancer cell lines caused activation of IFN signaling, accumulation of R-loops and DNA damage (85).

Induction of viral mimicry has great potential as a therapeutic approach for multiple cancers, including TNBC (86–89). In addition to the cell-intrinsic effects of activating innate immune pathways within the tumor, the signaling that occurs after activation of those pathways can promote antitumor immunity, especially when combined with checkpoint inhibitors (90–92). Combined therapies targeting ADAR1 and DHX9 may serve as an effective means of treating breast and potentially other cancers by inducing viral mimicry.

## Supplementary Material

Supplementary Tables Supplementary Tables

Source Data Figures Source Data Figures

Supplementary Figure 1 Supplementary Figure 1

Supplementary Figure 2 Supplementary Figure 2

Supplementary Figure 3 Supplementary Figure 3

Supplementary Figure 4 Supplementary Figure 4

Supplementary Figure 5 Supplementary Figure 5

Supplementary Figure 6 Supplementary Figure 6

Supplementary Figure 7 Supplementary Figure 7

Supplementary Figure 8 Supplementary Figure 8

Supplementary Figure 9 Supplementary Figure 9

Supplementary Figure 10 Supplementary Figure 10

Supplementary Figure 11 Supplementary Figure 11

Supplementary Figure 12 Supplementary Figure 12

Supplementary Figure 13 Supplementary Figure 13
