# Supplementary material for: Induction of Viral Mimicry Upon Loss of DHX9 and ADAR1 in Breast Cancer Cells
Source: Cancer Res Commun. 2024 Apr 4;4(4):986–1003. doi: 10.1158/2767-9764.CRC-23-0488 (PMC10993856; doi:10.1158/2767-9764.CRC-23-0488)

**Source Data Figures for:**

**Induction of viral mimicry upon loss of DHX9 and ADAR1 in breast cancer cells**

Kyle A. Cottrell ^1,4,5‡*^, Sua Ryu ^1,4‡^, Jackson R. Pierce ^5^, Luisangely Soto Torres ^1,4^, Holly E. Bohlin ^5^, Angela M. Schab ^1,4^, Jason D. Weber ^1,2,3,4*^

^1^Department of Medicine, Division of Molecular Oncology, ^2^Department of Cell Biology and Physiology, and ^3^Department of Biology, Siteman Cancer Center, ^4^ICCE Institute, Washington University School of Medicine, Saint Louis, Missouri, USA

^5^Department of Biochemistry, Purdue University, West Lafayette, IN, USA

^‡^ Authors contributed equally

*Co-Corresponding authors

Correspondence:

Kyle A Cottrell, Ph.D.

Department of Biochemistry

Purdue University

201 S University St.

West Lafayette, IN

Email: cottrellka@pudue.edu

Telephone: 765-494-6941

Jason D. Weber, Ph.D.

Department of Medicine

Division of Molecular Oncology

Washington University School of Medicine

660 South Euclid Avenue

Campus Box 8069

St. Louis, MO 63110 USA

Email: jweber@wustl.edu

Telephone: 314-747-3896

Fax: 314-362-0152

Below are the source data figures.


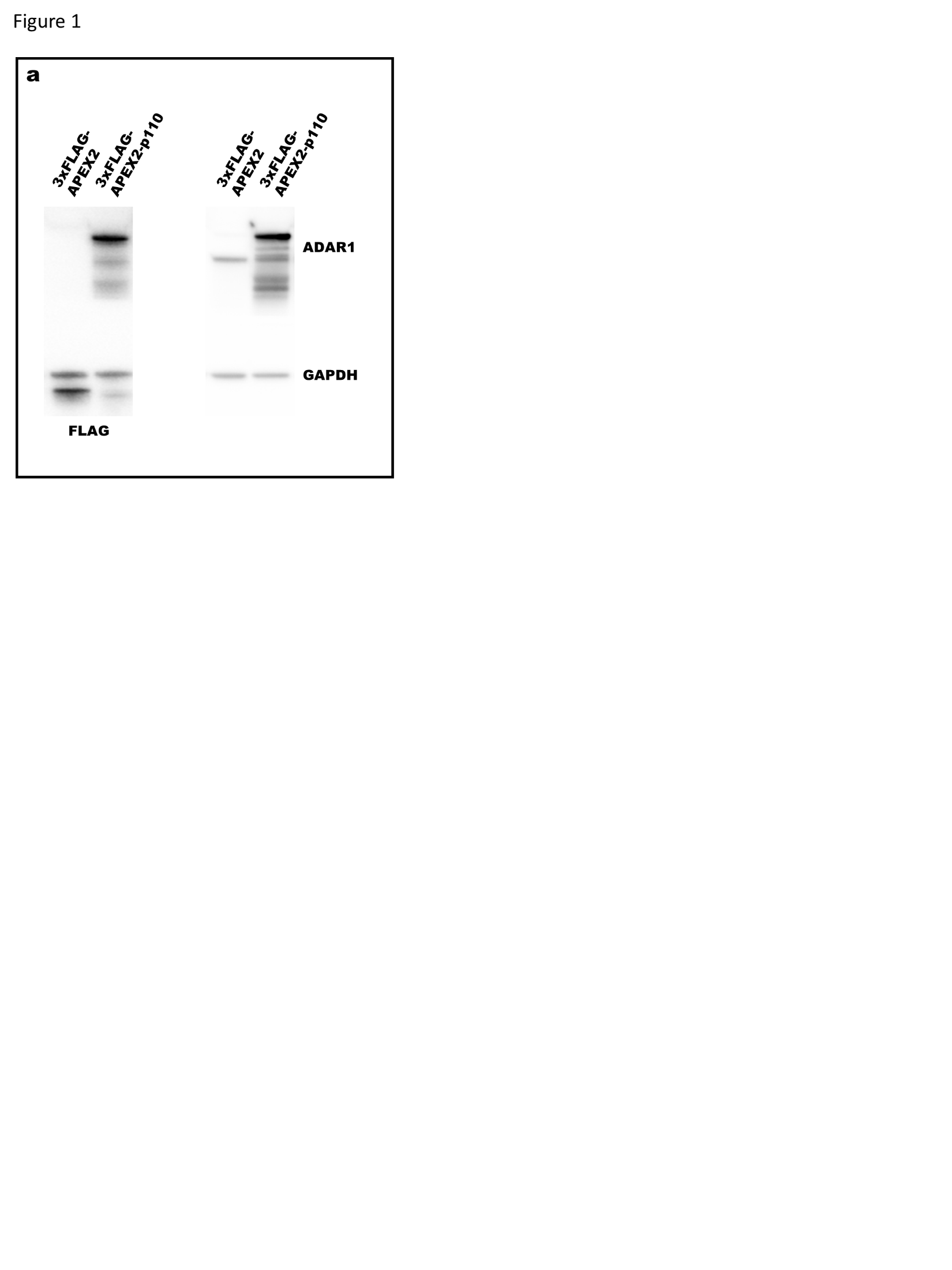


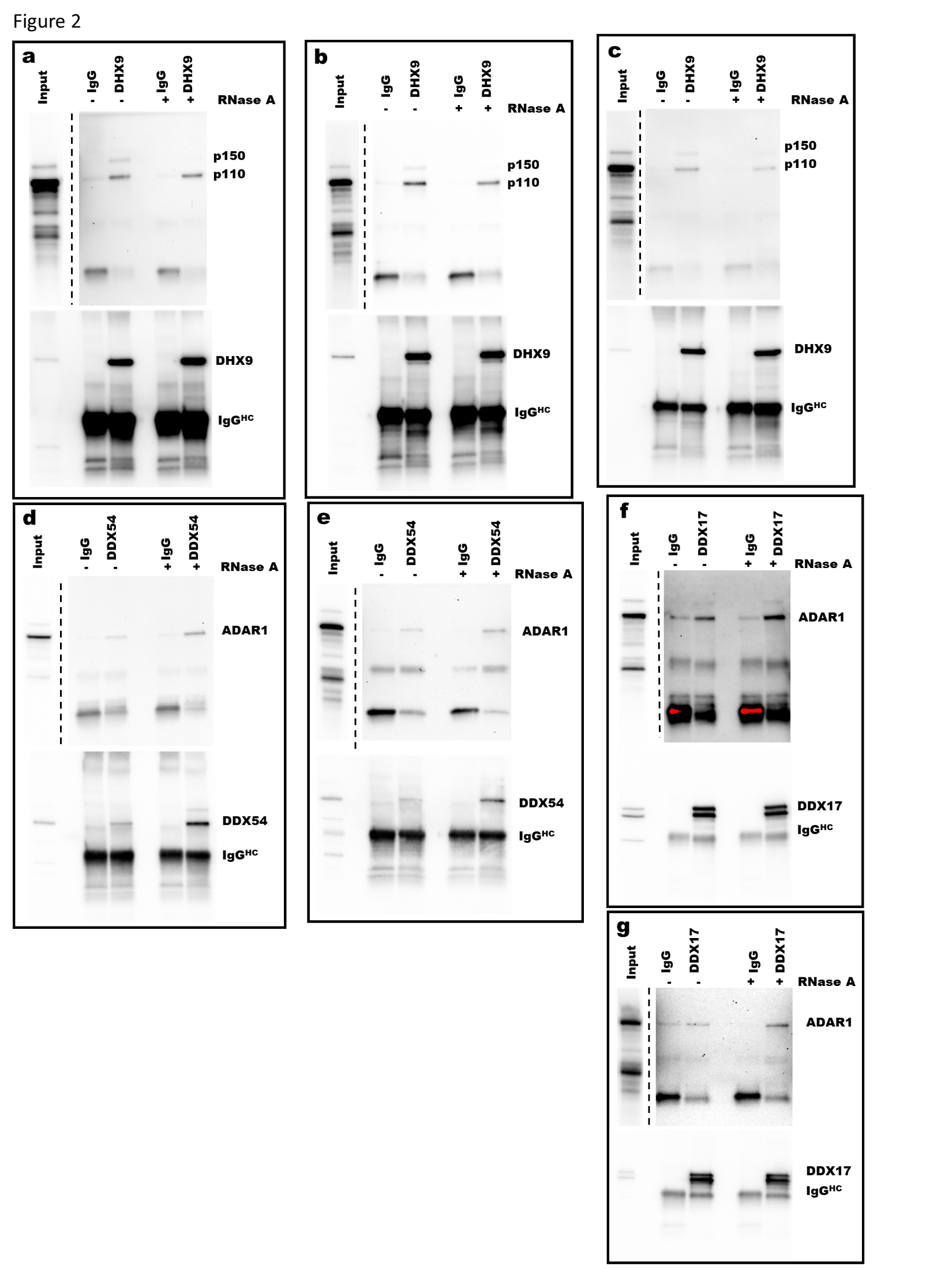


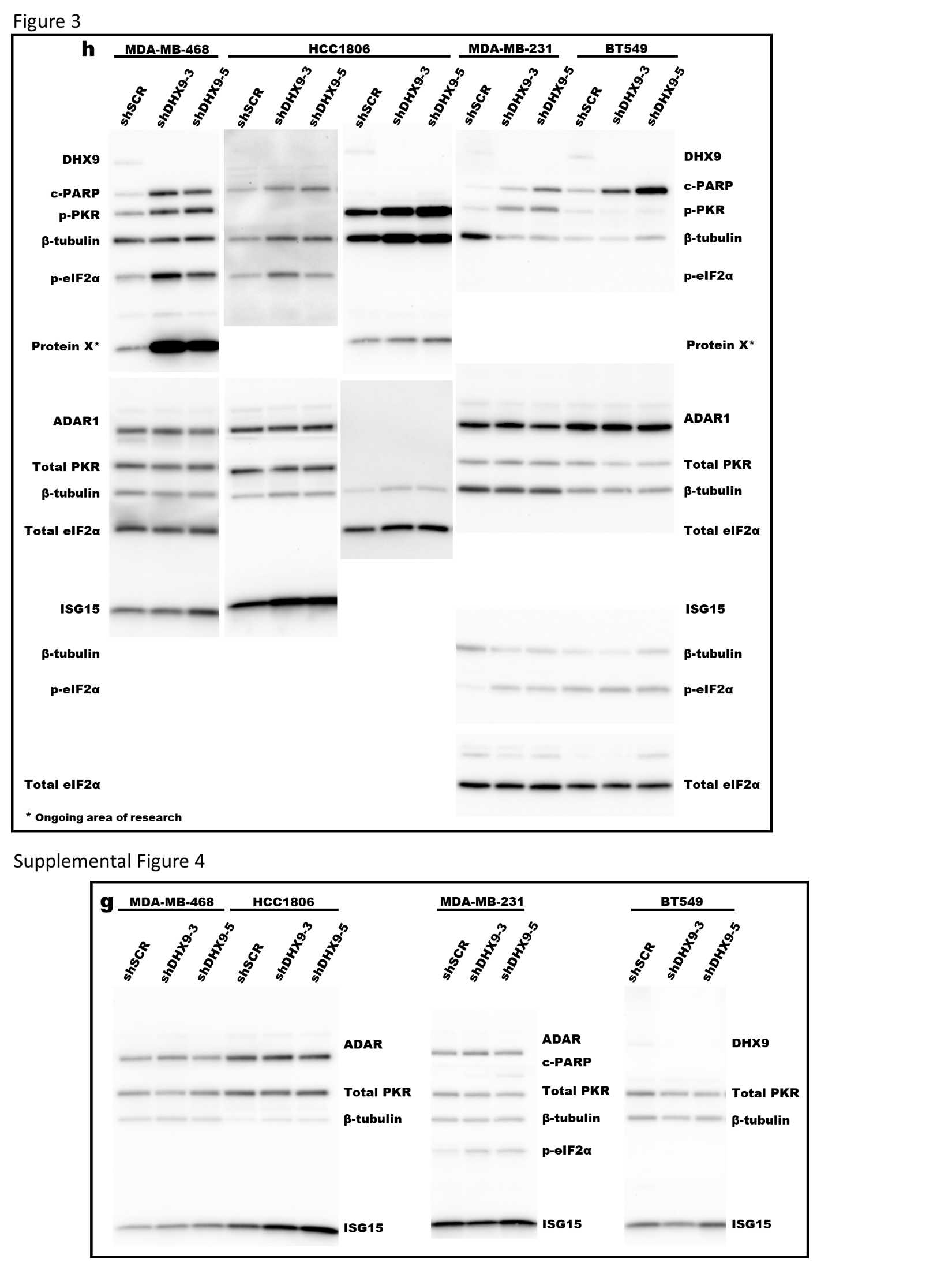


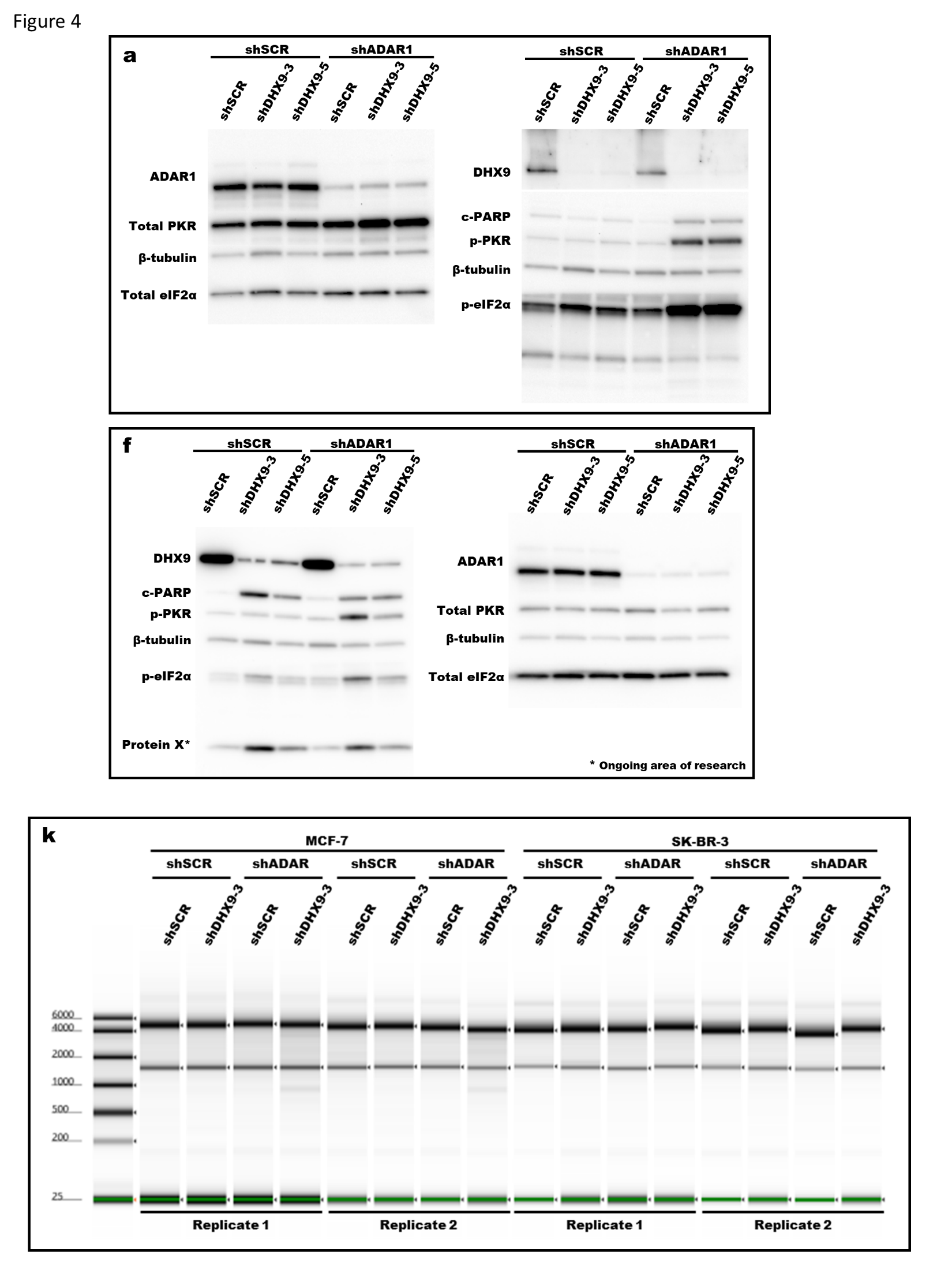


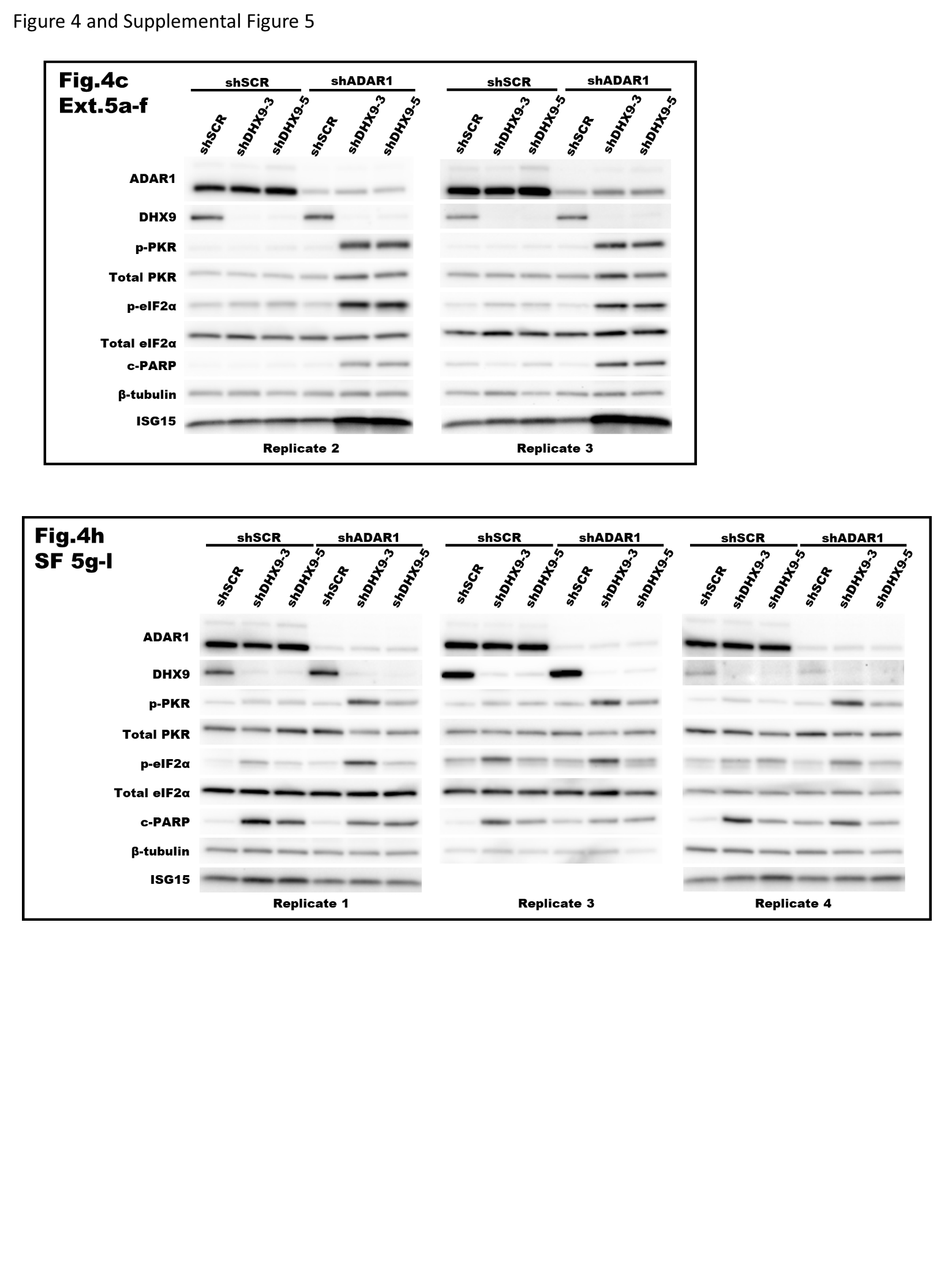


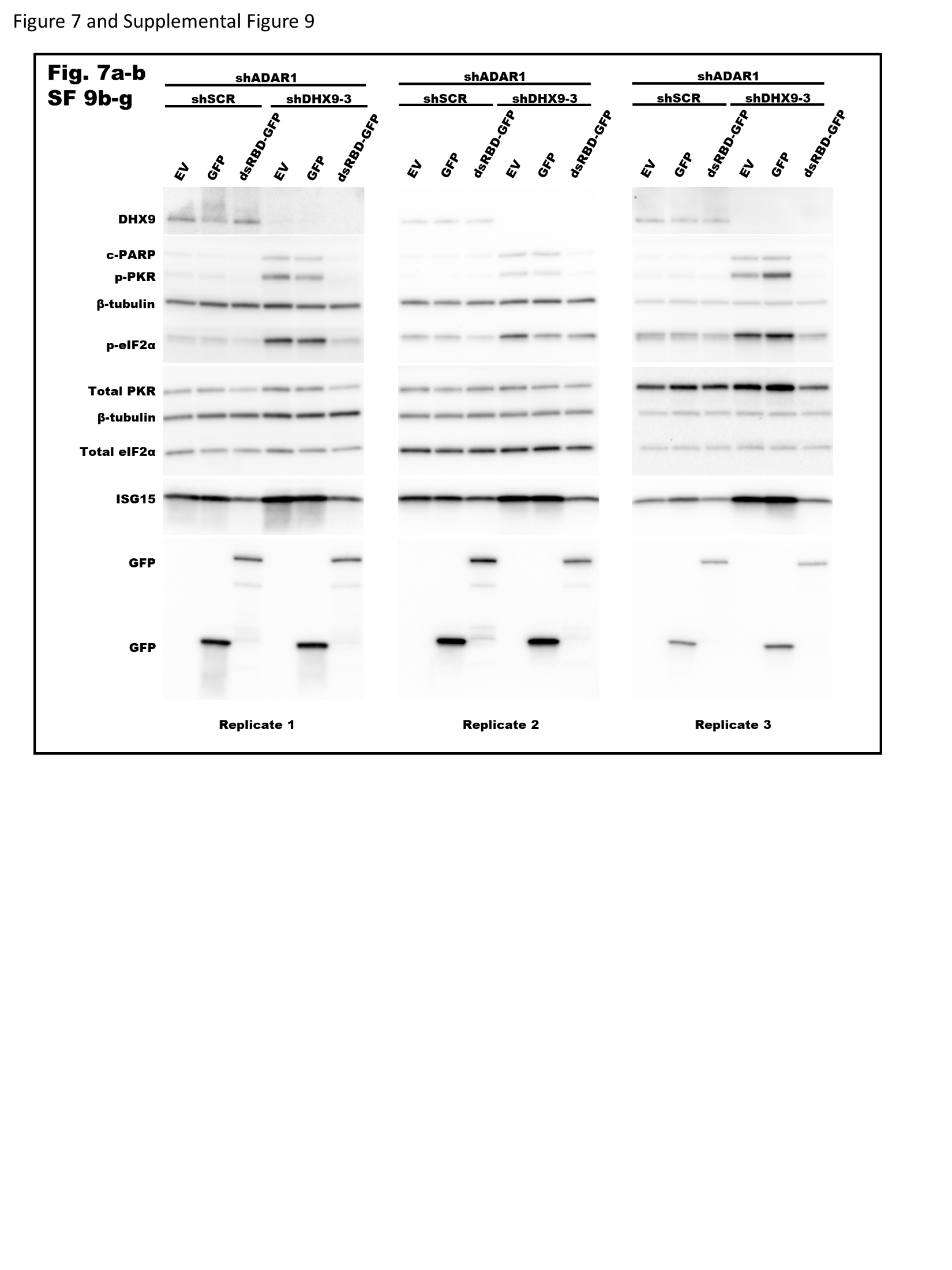


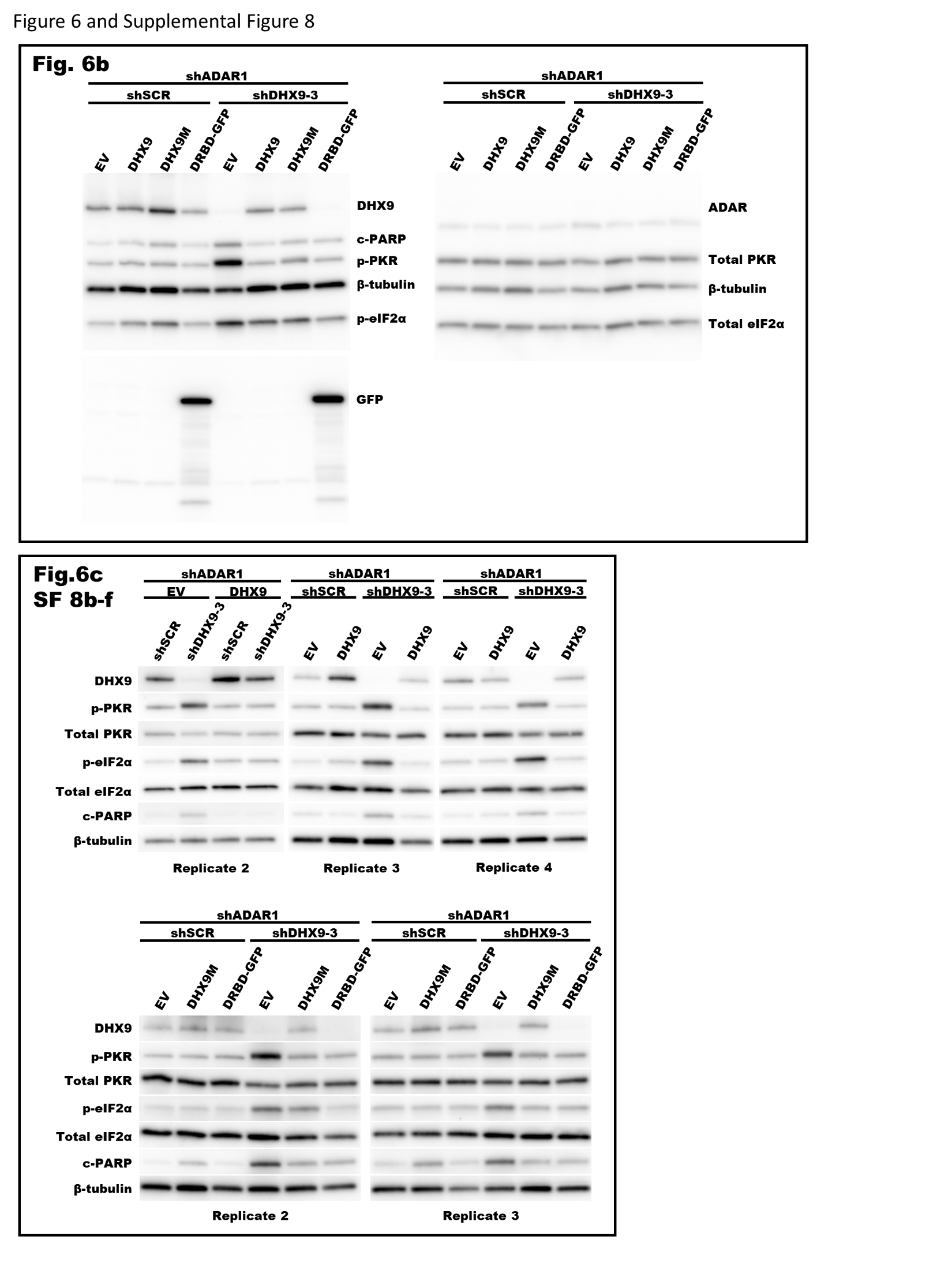


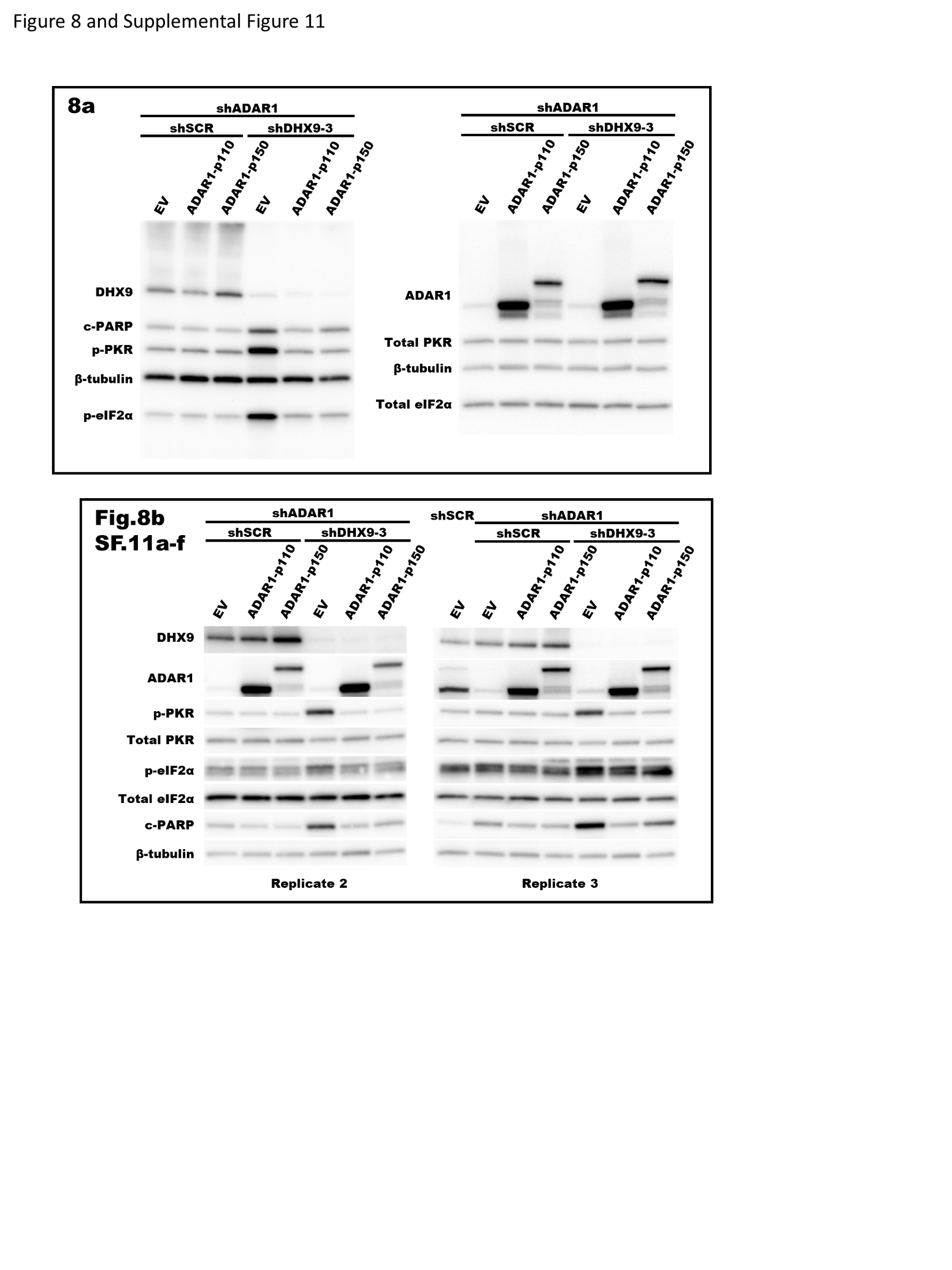


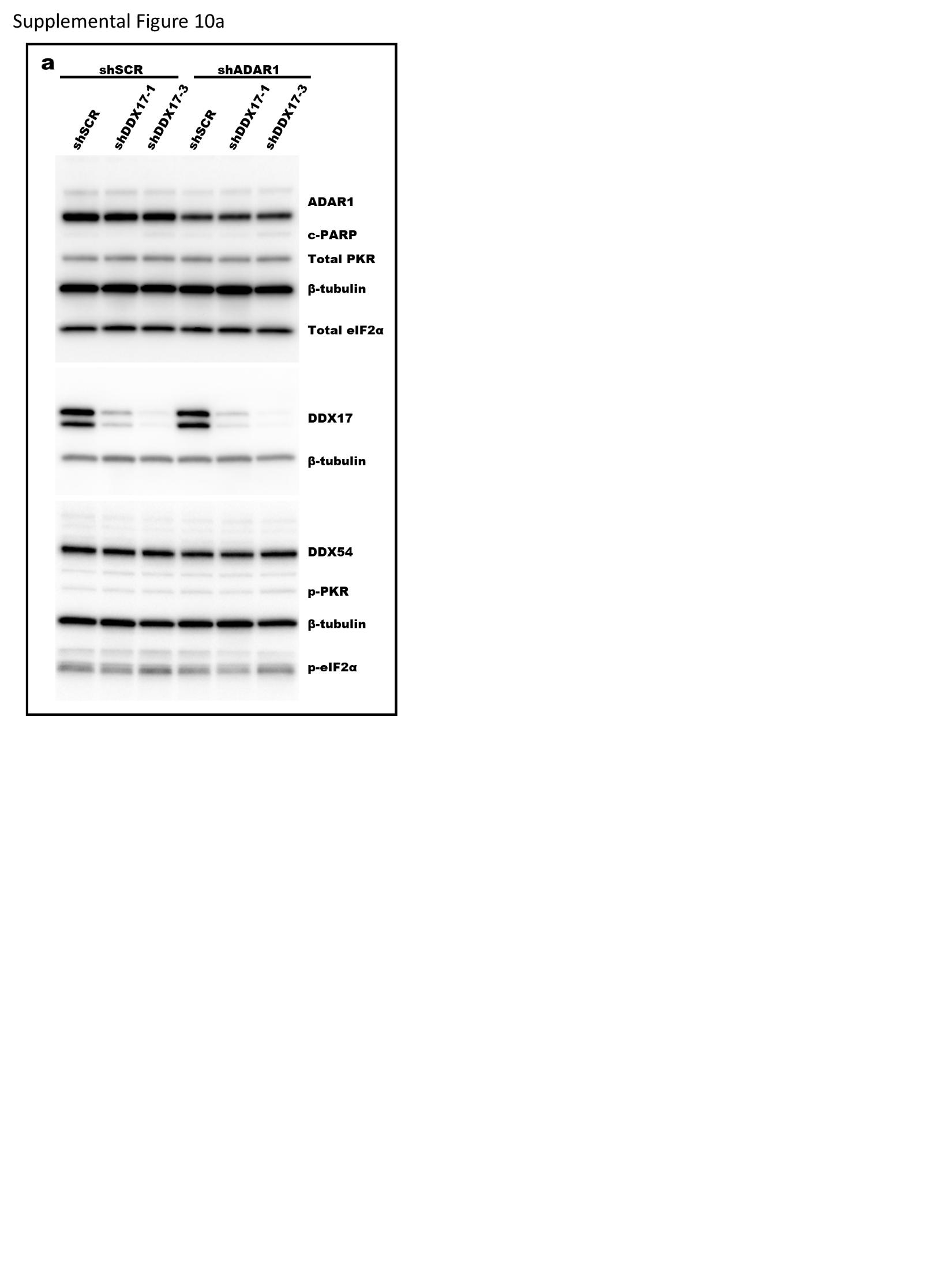

Supplement: Source Data Figures [file crc-23-0488-s02.docx]
