# Supplementary material for: Induction of Viral Mimicry Upon Loss of DHX9 and ADAR1 in Breast Cancer Cells
Source: Cancer Res Commun. 2024 Apr 4;4(4):986–1003. doi: 10.1158/2767-9764.CRC-23-0488 (PMC10993856; doi:10.1158/2767-9764.CRC-23-0488)
Supplement: Supplementary Figure 1 [file crc-23-0488-s03.pdf]

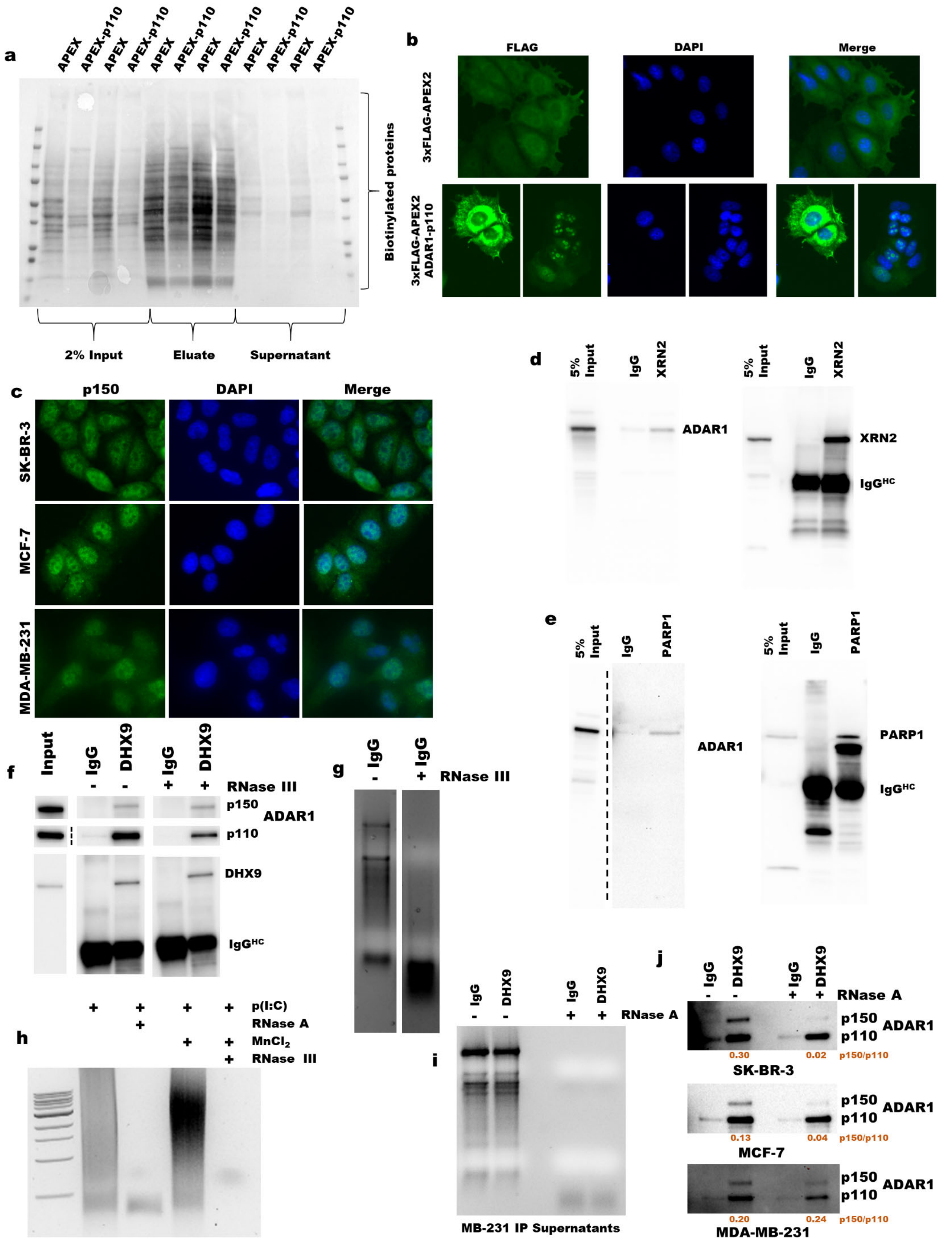
a
APEX
APEX-p110
APEX
APEX-p110
APEX
APEX-p110
APEX
APEX-p110
APEX
APEX-p110
APEX
APEX-p110
Biotinylated proteins
2% Input
Eluate
Supernatant
b
FLAG
DAPI
Merge
3xFLAG-APEX2
3xFLAG-APEX2
ADAR1-p110
c
p150
DAPI
Merge
SK-BR-3
MCF-7
MDA-MB-231
d
5% Input
IgG
XRN2
ADAR1
5% Input
IgG
XRN2
XRN2
IgG^HC
e
5% Input
IgG
PARP1
ADAR1
5% Input
IgG
PARP1
PARP1
IgG^HC
f
Input
IgG
DHX9
IgG
DHX9
- - + +
RNase III
p150
ADAR1
p110
DHX9
IgG^HC
g
- IgG
+ IgG
RNase III
h
p(I:C)
RNase A
MnCl2
RNase III
i
- IgG
- DHX9
+ IgG
+ DHX9
RNase A
MB-231 IP Supernatants
j
- IgG
- DHX9
+ IgG
+ DHX9
RNase A
p150
p110
ADAR1
0.30
0.02
p150/p110
SK-BR-3
p150
p110
ADAR1
0.13
0.04
p150/p110
MCF-7
p150
p110
ADAR1
0.20
0.24
p150/p110
MDA-MB-231

Figure S1:

a Representative streptavidin-HRP blot of the streptavidin pulldown for MCF-7. The '2% Input' represents the input used for the streptavidin pulldown following proximity labeling. The 'Eluate' is what was eluted from the streptavidin beads following pulldown. The 'Supernatant' is the supernatant following binding of the lysate to the streptavidin beads and before washing the beads. APEX refers to 3xFLAG-APEX2 and APEX-p110 refers to 3xFLAG-ADAR1-p110. **b** Immunofluorescence of 3xFLAG-APEX2 and 3xFLAG-ADAR1-p110 in MCF-7. Two representative images are shown for the APEX2-p110 fusion protein as the localization varied between cells within the polyclonal line used for labeling. **c** Indirect immunofluorescence for ADAR1-p150 in three breast cancer cell lines. **d-f** Representative blots following immunoprecipitation of XRN2 (**d**), PARP1 (**e**) or DHX9 (**f**) in SK-BR-3. Input represents 5% of the lysate used for immunoprecipitation. The IgG lanes represent immunoprecipitation eluates from pulldown with anti-rabbit IgG antibody. The lanes labeled XRN2, PARP1 or DHX9 indicate the eluates from immunoprecipitation with antibodies against those proteins respectively. The IgG^{HC} label indicates the band corresponding to the IgG heavy chain from the antibody used for immunoprecipitation. For panel **f**, ADAR1 was detected with an antibody specific for ADAR1-p150 (labeled p150 in the panel) or antibody that detects both isoforms (the band for ADAR1-p110 is shown and labeled p110). Additionally, for panel **f** the immunoprecipitation was performed with or without RNase III treatment during overnight antibody binding. **g** Assessment of rRNA integrity following immunoprecipitation in SK-BR-3, panel **f**. RNA was purified from the immunoprecipitation supernatants. rRNA integrity was assessed by denaturing agarose gel electrophoresis and staining with ethidium bromide. **h** Assessment of dsRNA degradation activity of RNase A and RNase III in the RIPA buffer used for immunoprecipitation. High-molecular weight p(I:C) was incubated overnight at 4 °C in the presence or absence of RNase A or RNase III ($MnCl_2$ was added to the RNase III treated sample and control). RNA was purified following treatment by phenol:chloroform extraction and ethanol precipitation. Purified RNA was assessed by agarose gel electrophoresis and staining with SYBR-Safe. The ladder is a 1 kB DNA ladder. **i** Assessment of rRNA integrity following immunoprecipitation as described in **g**, supernatants from DHX9 immunoprecipitation using lysates from MDA-MB-231 (Figure 2C) were assessed. **j** Digitally enhanced images of DHX9 immunoprecipitation immunoblots from Figure 2A-2C highlighting the presence of p150 in the eluates. The values colored orange are the ratio of ADAR1-p150 to ADAR1-p110 intensity for each representative blot.
