# Supplementary material for: Induction of Viral Mimicry Upon Loss of DHX9 and ADAR1 in Breast Cancer Cells
Source: Cancer Res Commun. 2024 Apr 4;4(4):986–1003. doi: 10.1158/2767-9764.CRC-23-0488 (PMC10993856; doi:10.1158/2767-9764.CRC-23-0488)
Supplement: Supplementary Figure 2 [file crc-23-0488-s04.pdf]

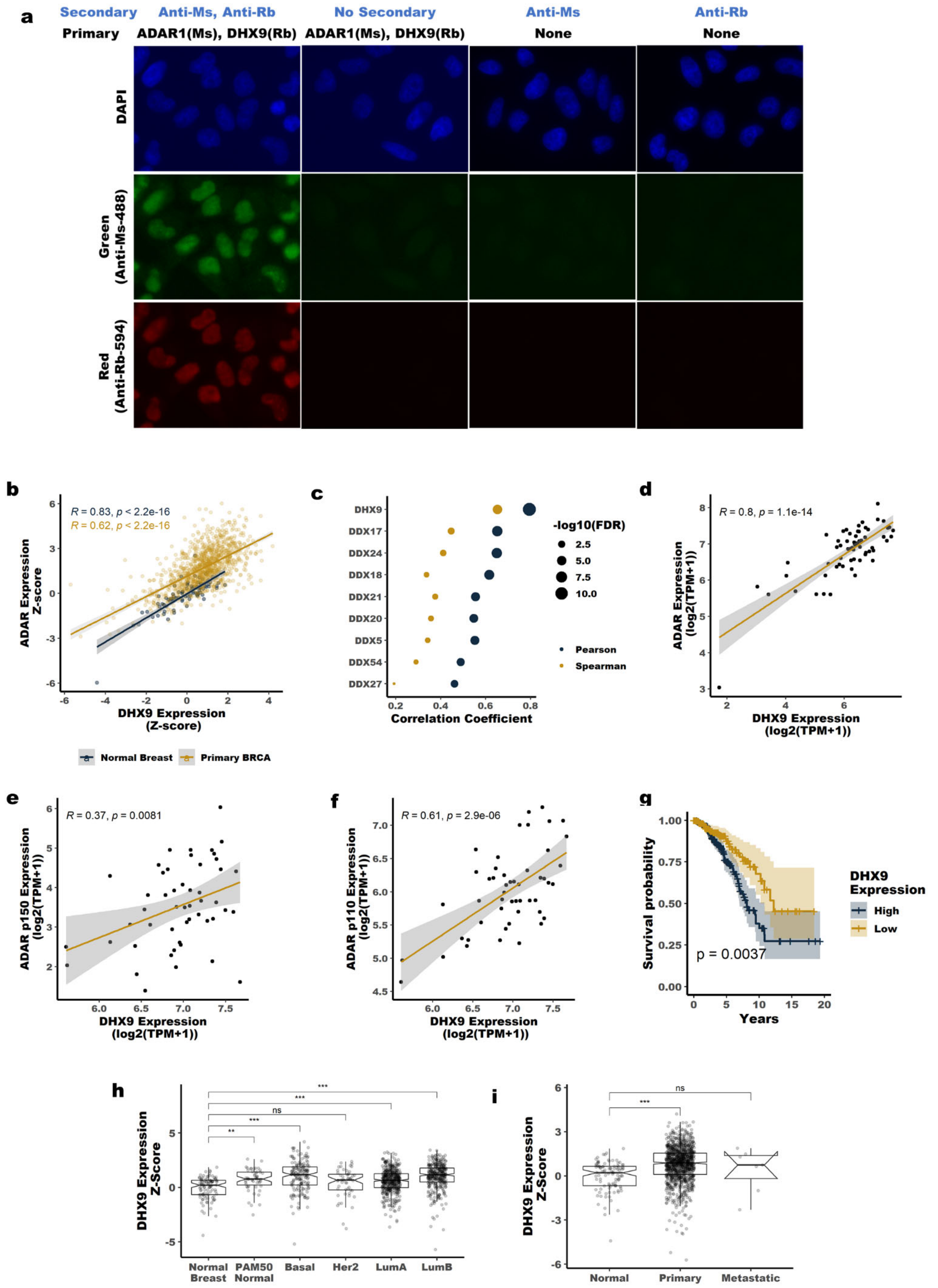
a
Secondary
Primary
Anti-Ms, Anti-Rb
ADAR1(Ms), DHX9(Rb)
No Secondary
ADAR1(Ms), DHX9(Rb)
Anti-Ms
None
Anti-Rb
None
DAPI
Green (Anti-Ms-488)
Red (Anti-Rb-594)
b
R = 0.83, p < 2.2e-16
R = 0.62, p < 2.2e-16
ADAR Expression Z-score
DHX9 Expression (Z-score)
Normal Breast
Primary BRCA
c
DHX9
DDX17
DDX24
DDX18
DDX21
DDX20
DDX5
DDX54
DDX27
Correlation Coefficient
-log10(FDR)
2.5
5.0
7.5
10.0
Pearson
Spearman
d
R = 0.8, p = 1.1e-14
ADAR Expression (log2(TPM+1))
DHX9 Expression (log2(TPM+1))
e
R = 0.37, p = 0.0081
ADAR p150 Expression (log2(TPM+1))
DHX9 Expression (log2(TPM+1))
f
R = 0.61, p = 2.9e-06
ADAR p110 Expression (log2(TPM+1))
DHX9 Expression (log2(TPM+1))
g
Survival probability
p = 0.0037
Years
DHX9 Expression
High
Low
h
DHX9 Expression Z-Score
Normal Breast
PAM50 Normal
Basal
Her2
LumA
LumB
**
***
ns
***
***
i
DHX9 Expression Z-Score
Normal
Primary
Metastatic
***
ns

Figure S2:

a Immunofluorescence controls for antibodies used in this study. Indirect immunofluorescence was performed for ADAR1 and DHX9 in SK-BR-3. Each column includes a different combination of primary and secondary antibodies. Ms = mouse, Rb = rabbit. **b** Scatter plot showing the correlation between ADAR1 and DHX9 expression at the RNA level in normal breast and primary BRCA, data from TCGA. The Pearson correlation coefficient and p-value are shown. **c** Pearson and Spearman correlation coefficients for the correlation between ADAR1 expression at the RNA level and the expression of each indicated helicase at the RNA level, data from breast cancer cell lines within CCLE. **d** Scatter plot showing the correlation between ADAR1 and DHX9 expression at the RNA level in breast cancer cell lines, data from CCLE. The Pearson correlation coefficient and p-value are shown. Scatterplots showing the correlation between ADAR1-p150 **e**, or ADAR1-p110 **f**, and DHX9 expression in breast cancer cell lines, data from CCLE. The Pearson correlation coefficient and p-value are shown. **g** Survival of breast cancer patients stratified by DHX9 expression. See methods for information on how the DHX9 expression cutoff was determined. Data from TCGA. **h** and **i** Expression of DHX9 at the RNA level based on PAM50 classification **h** or tumor site **i**, data from TCGA. * $p < 0.05$, ** $p < 0.01$, *** $p < 0.001$. P-values determined by Dunnett's test.
