# Supplementary material for: Induction of Viral Mimicry Upon Loss of DHX9 and ADAR1 in Breast Cancer Cells
Source: Cancer Res Commun. 2024 Apr 4;4(4):986–1003. doi: 10.1158/2767-9764.CRC-23-0488 (PMC10993856; doi:10.1158/2767-9764.CRC-23-0488)
Supplement: Supplementary Figure 3 [file crc-23-0488-s05.pdf]

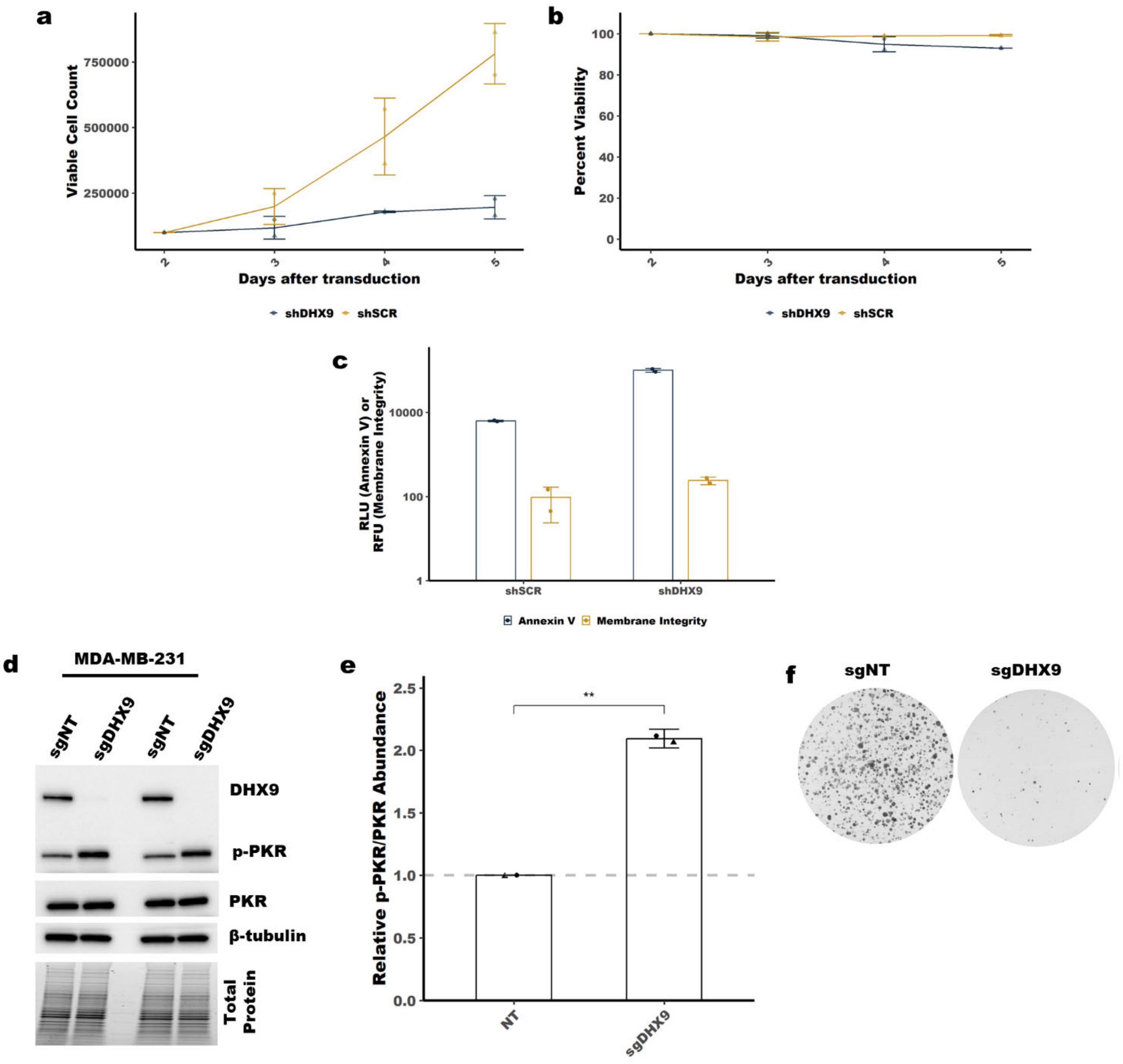

Figure S3:
a Cell count and **b** viability of cells as measured by trypan blue exclusion assay following knockdown of DHX9 in MDA-MB-231. **c** Assessment of apoptosis (Annexin V) and necroptosis (Membrane Integrity) by the Promega RealTime-Glo Apoptosis Assay, performed per manufactures instructions. Annexin V is measured by a NanoBit Luciferase system and is reported as Relative Luminescence Units (RLU). The necroptosis marker used in the kit is a fluorescent dye that labels cells with disrupted membrane integrity and is reported as Relative Fluorescence Units (RFU). **d** Immunoblot for DHX9, p-PKR, PKR and β-tubulin following knockout of DHX9 by CRISPR-Cas9 in MDA-MB-231, two replicates are shown, quantification of the p-PKR abundance is shown in **e**. **f** Representative foci formation assay results for knockout DHX9 in MDA-MB-231. Bars in panels **c** and **e** or the line in panels **a** and **b** represent the average of at two replicates, error bars are +/- SD. ** $p < 0.01$. P-values determined by Dunnett's test.
