# Supplementary material for: Induction of Viral Mimicry Upon Loss of DHX9 and ADAR1 in Breast Cancer Cells
Source: Cancer Res Commun. 2024 Apr 4;4(4):986–1003. doi: 10.1158/2767-9764.CRC-23-0488 (PMC10993856; doi:10.1158/2767-9764.CRC-23-0488)
Supplement: Supplementary Figure 4 [file crc-23-0488-s06.pdf]

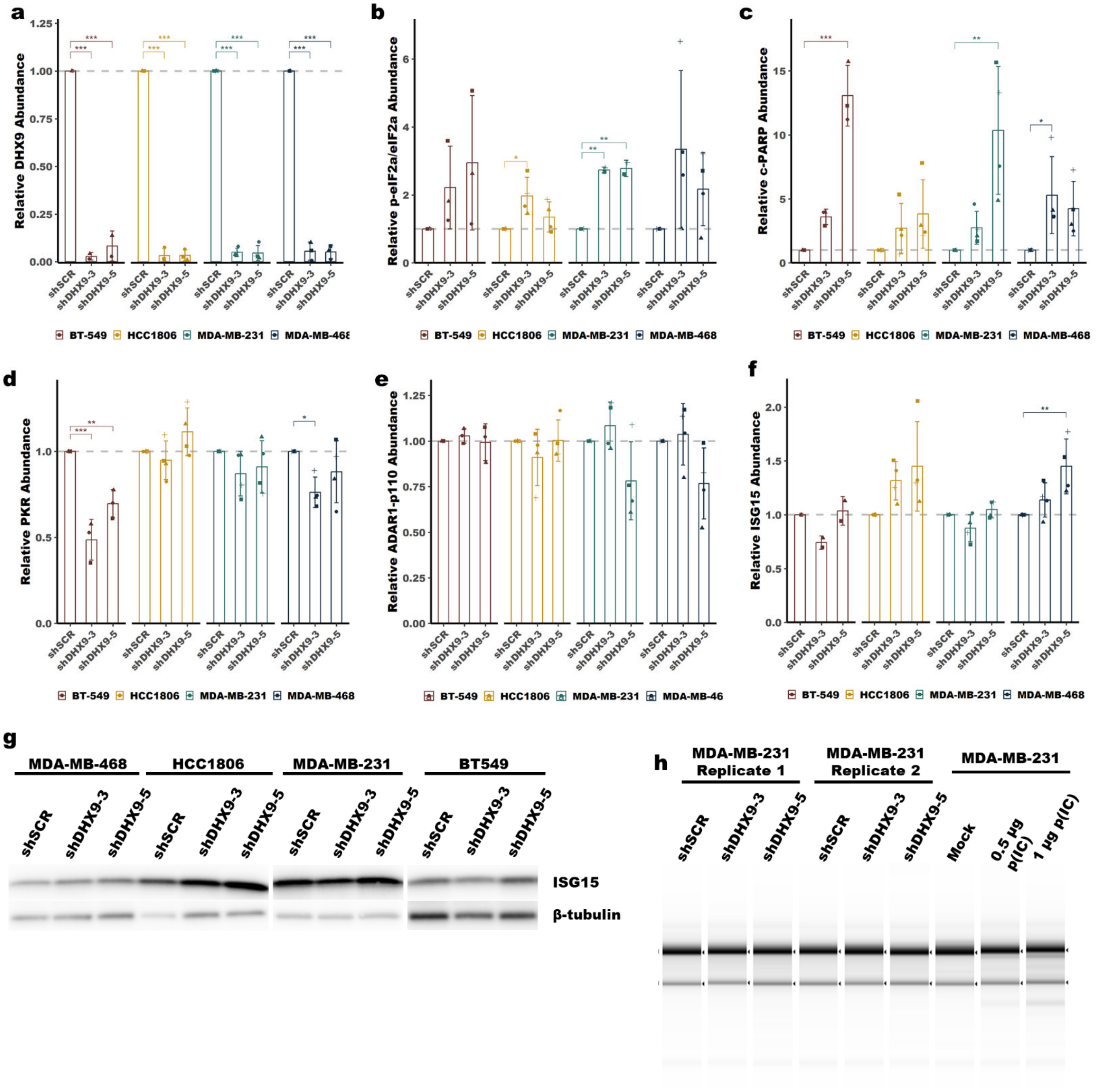

Figure S4:

a-e Quantification of the immunoblots shown in Figure 3g. **f** Quantification of the immunoblots shown in panel **g**. **g** Representative immunoblot for ISG15 and β-tubulin in TNBC cell lines following knockdown of DHX9. Immunoblots from additional experiments can be found in the Source Data Figures. **h** Assessment of rRNA integrity in MDA-MB-231 following knockdown of DHX9 or transfection with poly(I:C) (p(I:C)). Bars represent the average of at least three replicates **a-f**, error bars are +/- SD. * p <0.05, ** p <0.01, *** p < 0.001. P-values determined by Dunnett's test.
