# Supplementary material for: Induction of Viral Mimicry Upon Loss of DHX9 and ADAR1 in Breast Cancer Cells
Source: Cancer Res Commun. 2024 Apr 4;4(4):986–1003. doi: 10.1158/2767-9764.CRC-23-0488 (PMC10993856; doi:10.1158/2767-9764.CRC-23-0488)
Supplement: Supplementary Figure 5 [file crc-23-0488-s07.pdf]

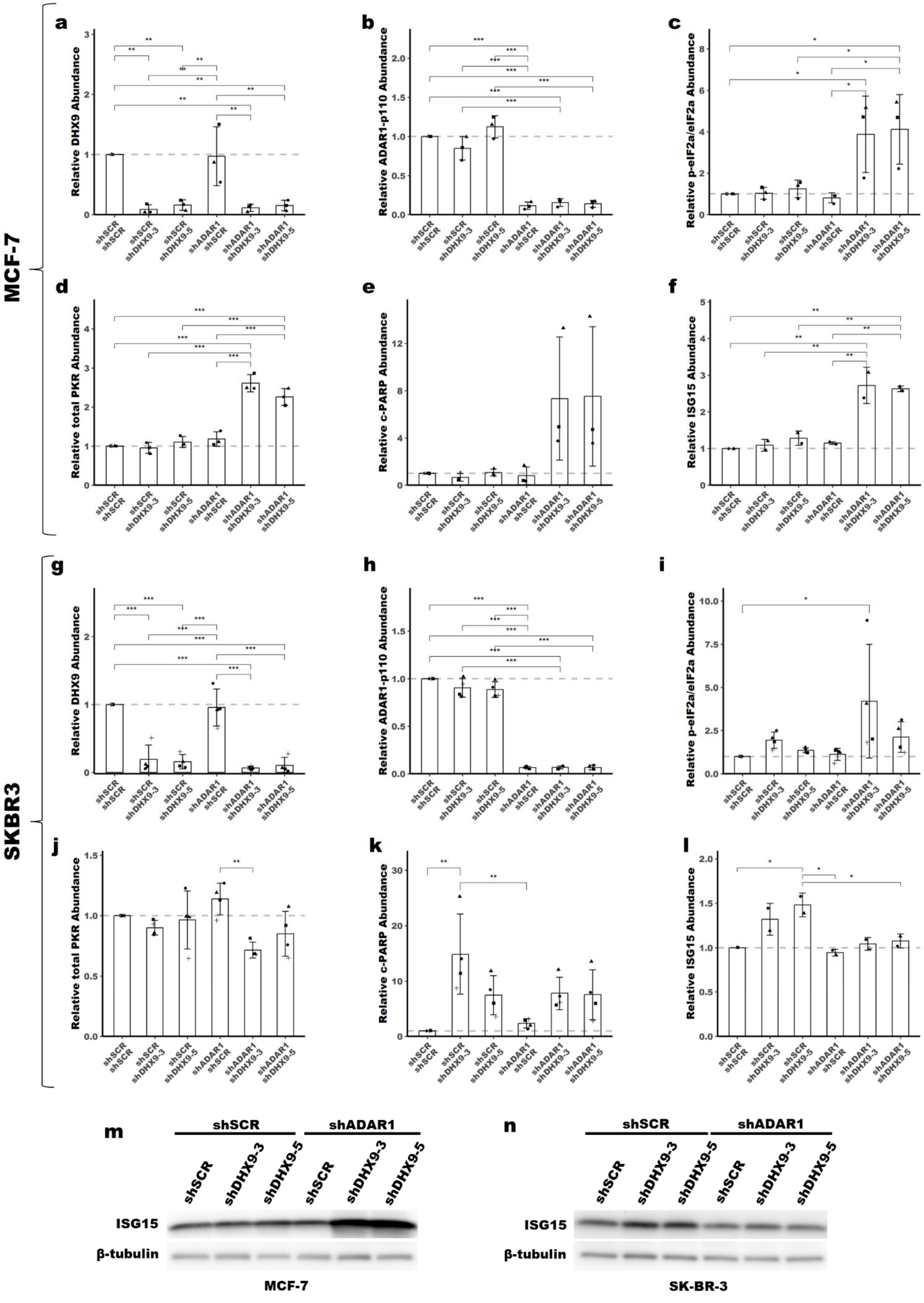
MCF-7
SKBR3
a
Relative DHX9 Abundance
b
Relative ADAR1-p110 Abundance
c
Relative p-eIF2a/eIF2a Abundance
d
Relative total PKR Abundance
e
Relative c-PARP Abundance
f
Relative ISG15 Abundance
g
Relative DHX9 Abundance
h
Relative ADAR1-p110 Abundance
i
Relative p-eIF2a/eIF2a Abundance
j
Relative total PKR Abundance
k
Relative c-PARP Abundance
l
Relative ISG15 Abundance
shSCR shSCR
shSCR shDHX9-3
shSCR shDHX9-5
shADAR1 shSCR
shADAR1 shDHX9-3
shADAR1 shDHX9-5
m
shSCR
shADAR1
shSCR
shDHX9-3
shDHX9-5
shSCR
shDHX9-3
shDHX9-5
ISG15
β-tubulin
MCF-7
n
shSCR
shADAR1
shSCR
shDHX9-3
shDHX9-5
shSCR
shDHX9-3
shDHX9-5
ISG15
β-tubulin
SK-BR-3

Figure S5:

a-**l** Quantification of the immunoblots shown in Figure 4a, 4f and panels **m** and **n** in this figure. **m**-**n** Representative immunoblot for ISG15 and β-tubulin in MCF-7 and SK-BR-3 following knockdown of DHX9 and/or ADAR1. Immunoblots from additional experiments can be found in the Source Data Figures. Bars represent the average of at least three replicates **a**-**e** and **g**-**k** or two replicates **f** and **l**, error bars are +/- SD. * $p < 0.05$, ** $p < 0.01$, *** $p < 0.001$. P-values determined by one-way ANOVA with post-hoc Tukey. Comparisons between the two different shRNAs targeting DHX9 (shDHX9-3 and shDHX9-5) were not included for clarity.
