# Supplementary material for: Induction of Viral Mimicry Upon Loss of DHX9 and ADAR1 in Breast Cancer Cells
Source: Cancer Res Commun. 2024 Apr 4;4(4):986–1003. doi: 10.1158/2767-9764.CRC-23-0488 (PMC10993856; doi:10.1158/2767-9764.CRC-23-0488)
Supplement: Supplementary Figure 6 [file crc-23-0488-s08.pdf]

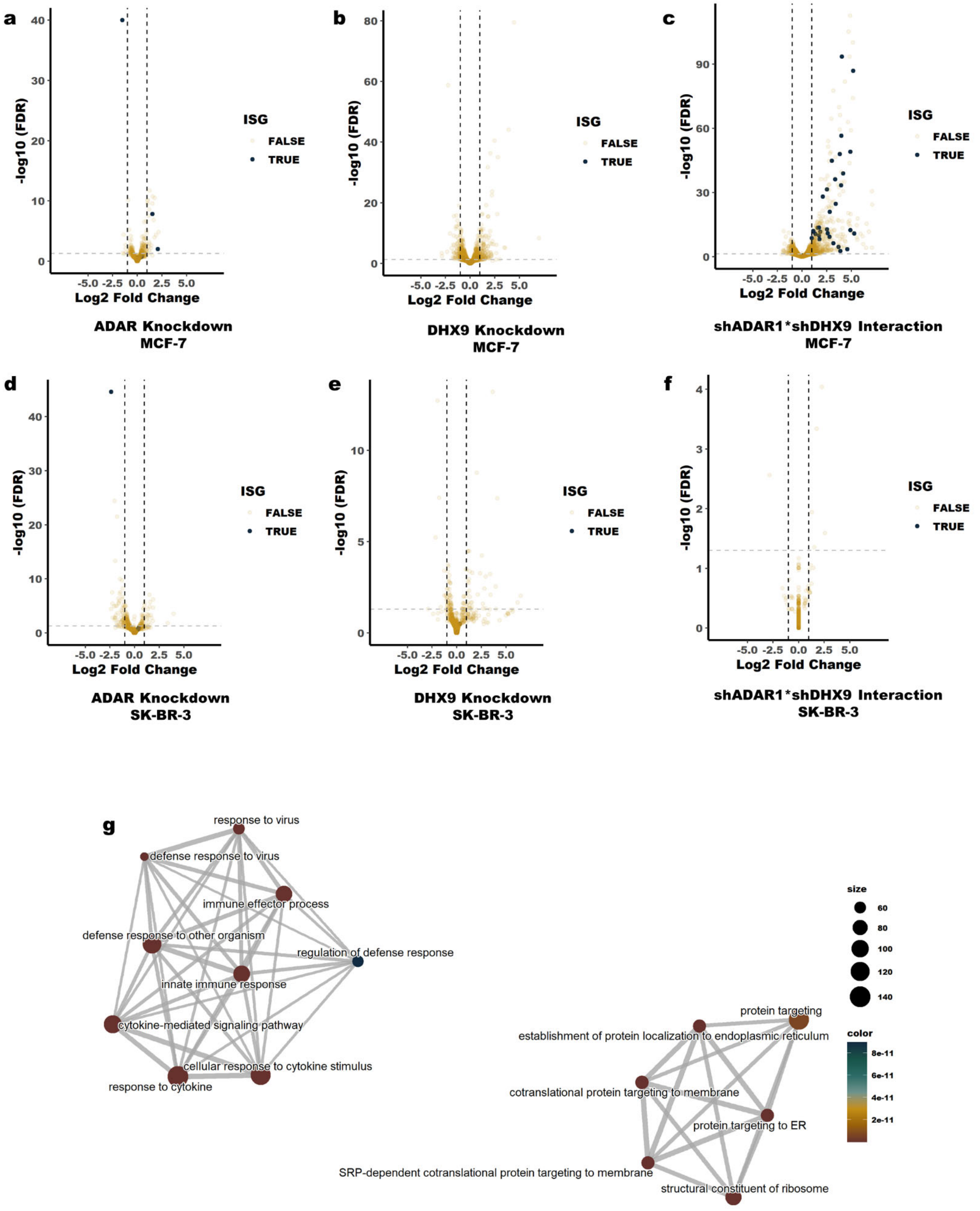

a
b
c
-log10 (FDR)
ISG
FALSE
TRUE
Log2 Fold Change
ADAR Knockdown
MCF-7
DHX9 Knockdown
MCF-7
shADAR1*shDHX9 Interaction
MCF-7
d
e
f
ADAR Knockdown
SK-BR-3
DHX9 Knockdown
SK-BR-3
shADAR1*shDHX9 Interaction
SK-BR-3
g
response to virus
defense response to virus
immune effector process
defense response to other organism
regulation of defense response
innate immune response
cytokine-mediated signaling pathway
cellular response to cytokine stimulus
response to cytokine
protein targeting
establishment of protein localization to endoplasmic reticulum
cotranslational protein targeting to membrane
protein targeting to ER
SRP-dependent cotranslational protein targeting to membrane
structural constituent of ribosome
size
60
80
100
120
140
color
8e-11
6e-11
4e-11
2e-11

Figure S6:

a-**f** Volcano plots showing changes in RNA expression upon knockdown of DHX9 and/or ADAR1 in MCF-7 or SK-BR-3. For panels **c** and **f**, fold-change of RNA expression was determined using an interaction term between ADAR1 and DHX9 knockdown. For all volcano plots, Core ISGs have been labeled. The ISG with lower expression upon knockdown of ADAR1 in MCF-7 and SK-BR-3 **a** and **d** is ADAR1. **g** Enrichment map for the GO terms described in Figure 5b and Supplementary Table 14 (based on combined knockdown of ADAR1 and DHX9 in MCF-7). The GO terms in the left cluster are upregulated following knockdown of DHX9 and ADAR1, while the terms in the right cluster are downregulated. The size of each point represents the number of genes associated with the GO term and the color is the FDR corrected p-value.
