# Supplementary material for: Induction of Viral Mimicry Upon Loss of DHX9 and ADAR1 in Breast Cancer Cells
Source: Cancer Res Commun. 2024 Apr 4;4(4):986–1003. doi: 10.1158/2767-9764.CRC-23-0488 (PMC10993856; doi:10.1158/2767-9764.CRC-23-0488)
Supplement: Supplementary Figure 7 [file crc-23-0488-s09.pdf]

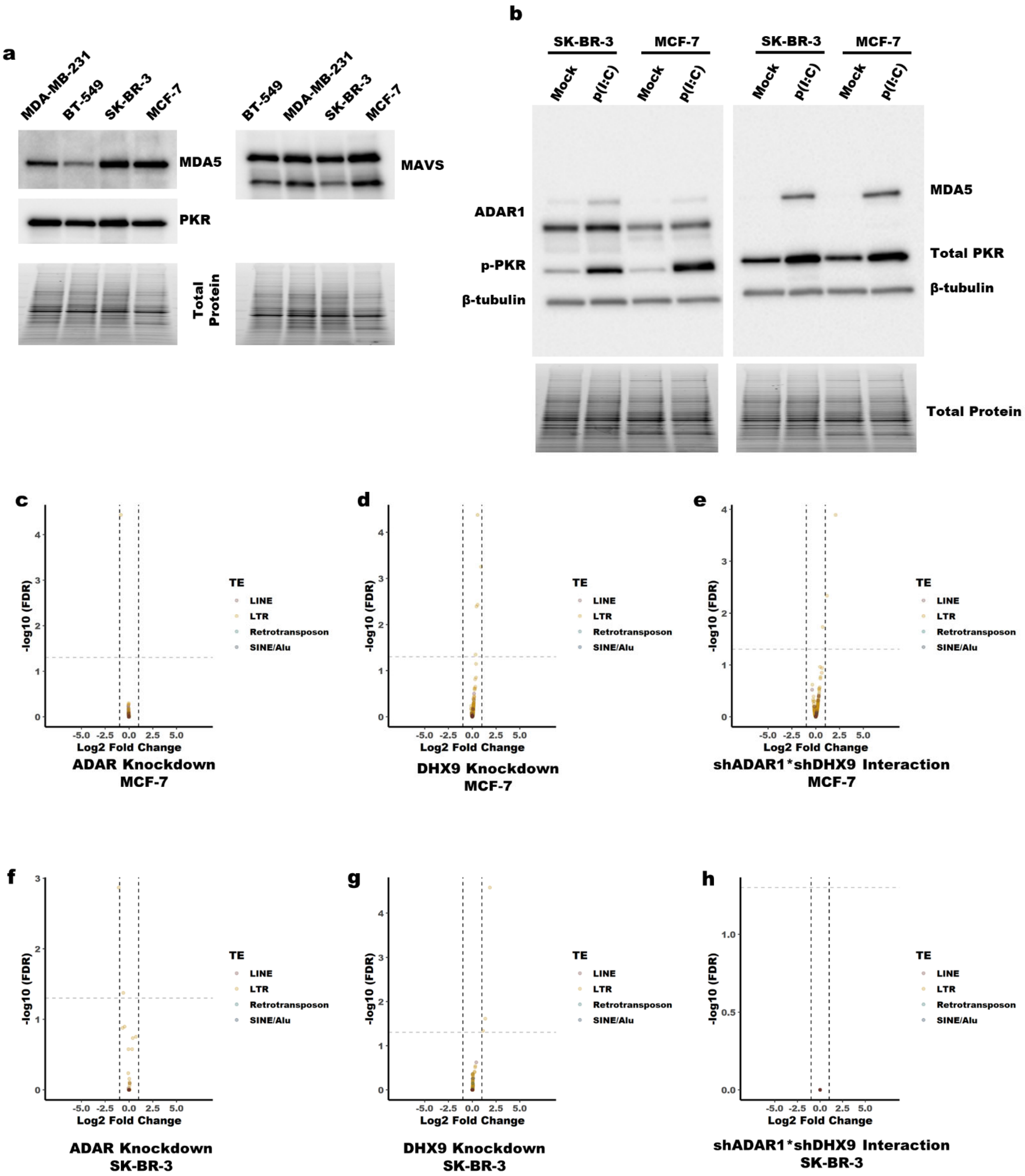

Figure S7:

a Immunoblot for MDA5, PKR and MAVS expression in breast cancer cell lines of interest. **b** Immunoblot for assessing activation of PKR and type I IFN signaling following transfection of MCF-7 or SK-BR-3 with p(I:C). **c-h** Volcano plots showing changes in transposable element expression upon knockdown of DHX9 and/or ADAR1 in MCF-7 or SK-BR-3. For panels **e** and **h**, fold-change of RNA expression was determined using an interaction term between ADAR1 and DHX9 knockdown. Individual points are colored based on the transposable element family. Retrotransposon refers to SINE-VNTR-Alus (SVA) retrotransposons. Data for differential expression for other types of transposable elements can be found in Supplemental Tables 18-23.
