# Supplementary material for: Induction of Viral Mimicry Upon Loss of DHX9 and ADAR1 in Breast Cancer Cells
Source: Cancer Res Commun. 2024 Apr 4;4(4):986–1003. doi: 10.1158/2767-9764.CRC-23-0488 (PMC10993856; doi:10.1158/2767-9764.CRC-23-0488)
Supplement: Supplementary Figure 9 [file crc-23-0488-s11.pdf]

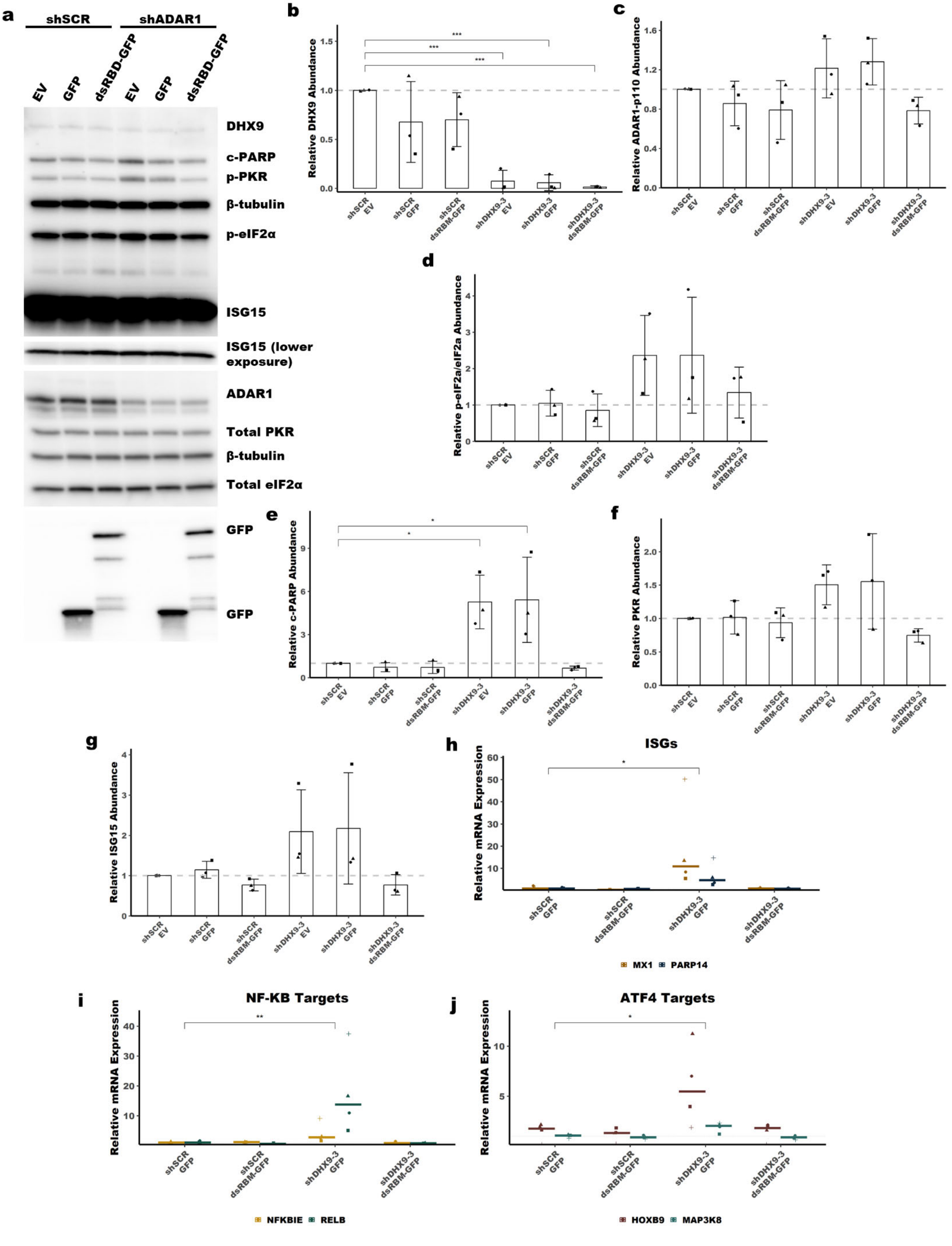
a
shSCR
shADAR1
EV
GFP
dsRBD-GFP
EV
GFP
dsRBD-GFP
DHX9
c-PARP
p-PKR
β-tubulin
p-eIF2α
ISG15
ISG15 (lower exposure)
ADAR1
Total PKR
β-tubulin
Total eIF2α
GFP
GFP
b
Relative DHX9 Abundance
***
***
***
c
Relative ADAR1-p110 Abundance
d
Relative p-eIF2a/eIF2a Abundance
e
Relative c-PARP Abundance
*
*
f
Relative PKR Abundance
shSCR EV
shSCR GFP
shSCR dsRBM-GFP
shDHX9-3 EV
shDHX9-3 GFP
shDHX9-3 dsRBM-GFP
g
Relative ISG15 Abundance
h
ISGs
Relative mRNA Expression
*
MX1
PARP14
i
NF-KB Targets
Relative mRNA Expression
**
NFKBIE
RELB
j
ATF4 Targets
Relative mRNA Expression
*
HOXB9
MAP3K8

Figure S9:

a Representative immunoblot showing the expression of the constructs used in the rescue experiments described in Figure 6. **b-g** Quantification of the immunoblot in Figure 6f. **h-j** qRT-PCR data shown in Figure 6h separated by pathway. Bars represent the average of at least three replicates, error bars are +/- SD. * $p < 0.05$, ** $p < 0.01$, *** $p < 0.001$. P-values determined by Dunnett's test.
