# Supplementary material for: Induction of Viral Mimicry Upon Loss of DHX9 and ADAR1 in Breast Cancer Cells
Source: Cancer Res Commun. 2024 Apr 4;4(4):986–1003. doi: 10.1158/2767-9764.CRC-23-0488 (PMC10993856; doi:10.1158/2767-9764.CRC-23-0488)
Supplement: Supplementary Figure 10 [file crc-23-0488-s12.pdf]

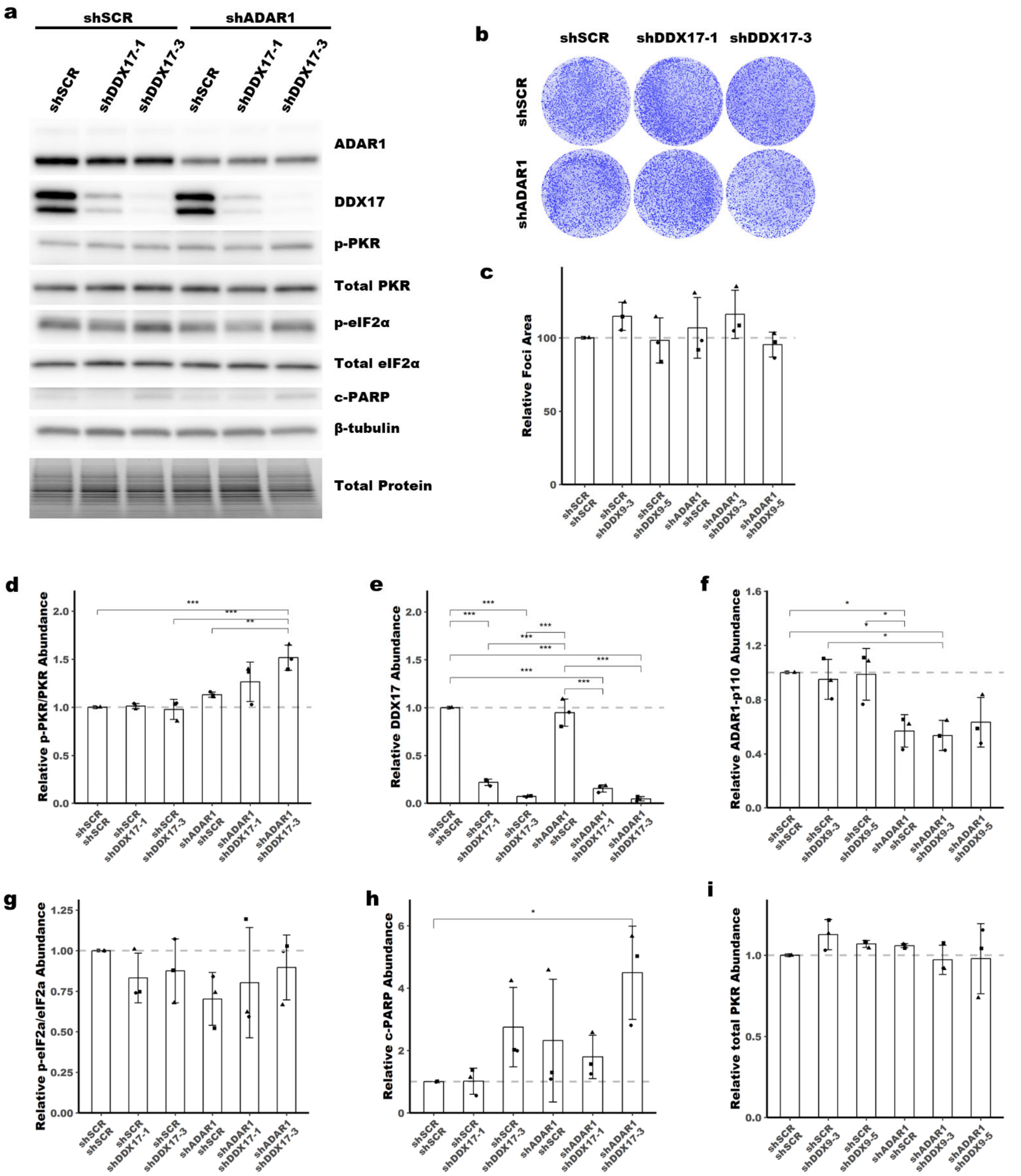

Figure S10:

a Representative immunoblot showing the phenotype of ADAR1 and/or DDX17 knockdown in SK-BR-3. Uncropped blots can be found in Source Data Figures. Protein abundance from the immunoblot in **a** was normalized by total protein abundance by quantification of the Stain Free gel image (bottom of panel). **b** Representative foci formation phenotype of ADAR1 and/or DD17 knockdown in SK-BR-3, quantification of relative foci area is shown in **c**. **d-i** Quantification of the immunoblot in **a**. Bars represent the average of at least three replicates, error bars are +/- SD. * $p < 0.05$, ** $p < 0.01$, *** $p < 0.001$.

Figure S10: (cont.)
P-values determined by one-way ANOVA with post-hoc Tukey. Comparisons between the two different shRNAs targeting DDX17 (shDDX17-1 and shDDX17-3) were not included for clarity.
