# Supplementary material for: Induction of Viral Mimicry Upon Loss of DHX9 and ADAR1 in Breast Cancer Cells
Source: Cancer Res Commun. 2024 Apr 4;4(4):986–1003. doi: 10.1158/2767-9764.CRC-23-0488 (PMC10993856; doi:10.1158/2767-9764.CRC-23-0488)
Supplement: Supplementary Figure 11 [file crc-23-0488-s13.pdf]

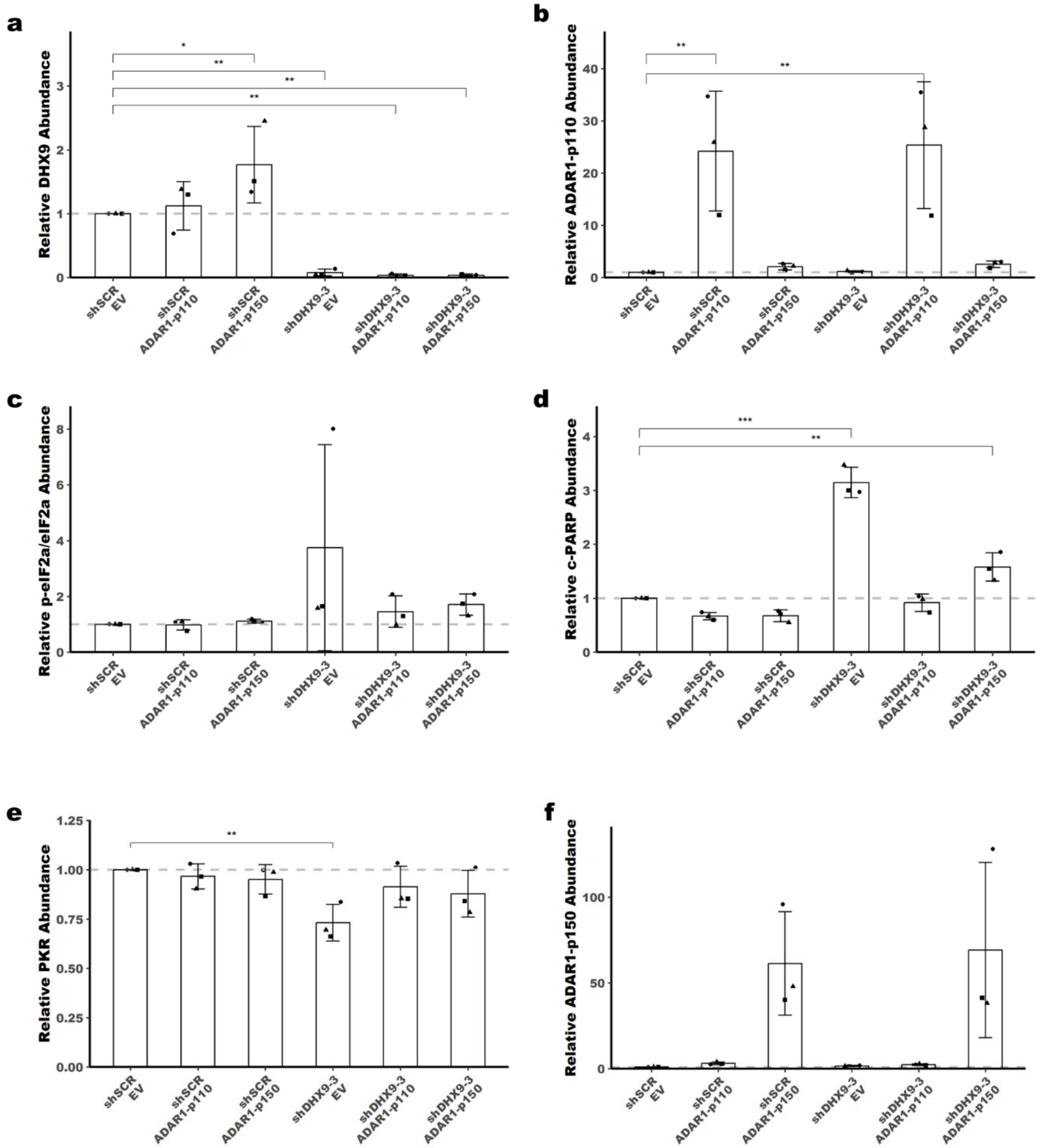


**Figure S11:**

**a-f** Quantification of the immunoblot in Figure 7a. Bars represent the average of at least three replicates, error bars are +/- SD. * p <0.05, ** p <0.01, *** p < 0.001. P-values determined by Dunnett's test.
