# Supplementary material for: Induction of Viral Mimicry Upon Loss of DHX9 and ADAR1 in Breast Cancer Cells
Source: Cancer Res Commun. 2024 Apr 4;4(4):986–1003. doi: 10.1158/2767-9764.CRC-23-0488 (PMC10993856; doi:10.1158/2767-9764.CRC-23-0488)
Supplement: Supplementary Figure 12 [file crc-23-0488-s14.pdf]

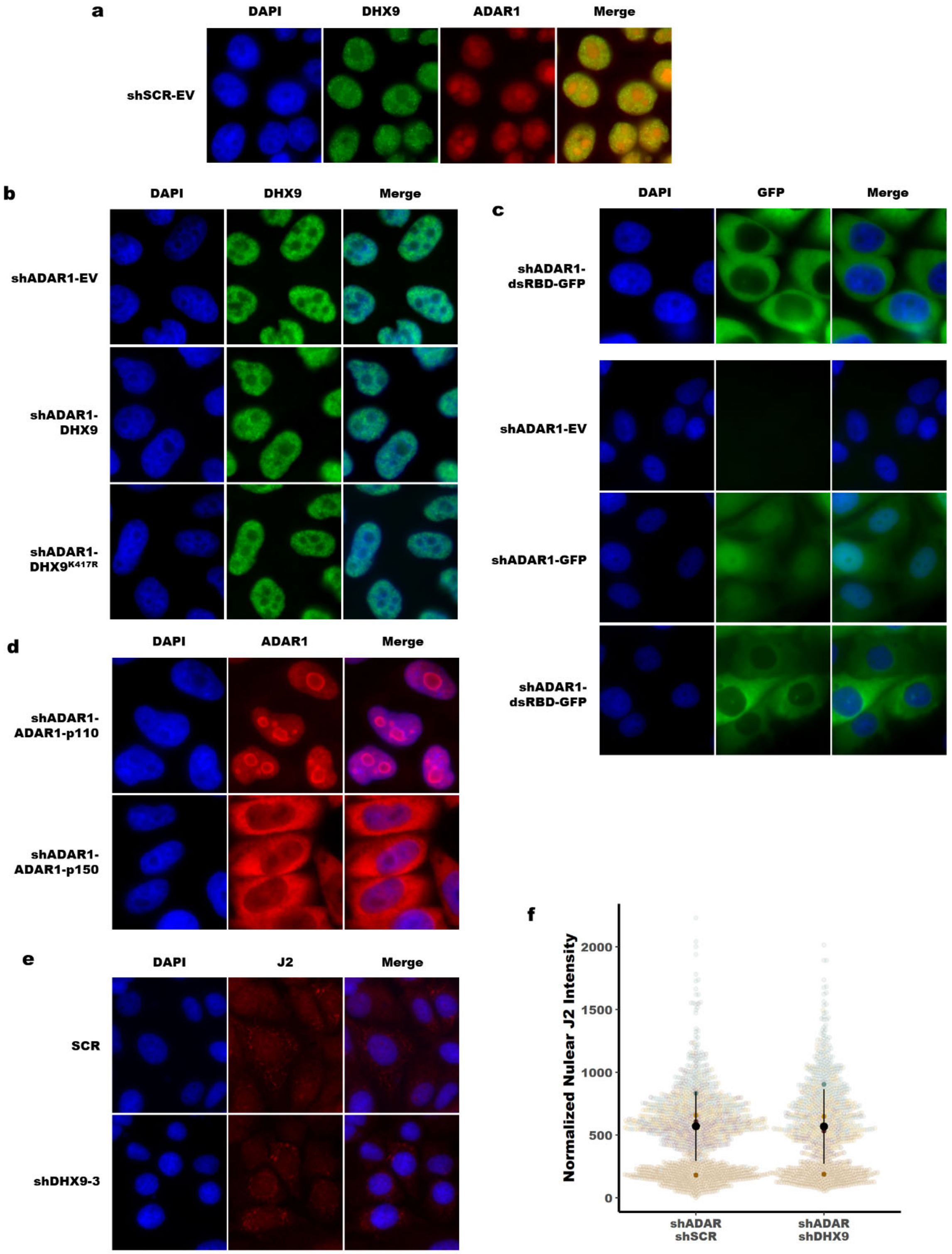

Figure S12:

Immunofluorescence for ADAR1 and DHX9 **a**, DHX9 **b**, GFP **c**, and ADAR1 **d** in SK-BR-3 infected with knockdown and overexpression constructs used in Figure 6. **e** Representative immunofluorescence for dsRNA with the J2 antibody following knockdown of ADAR1 (both conditions) and DHX9 (shDHX9-3). Nuclear J2 intensity is quantified for four replicates in panel **f**. The transparent colored dots (background) represent individual nuclei across three separate fields for each replicate. The opaque colored dots are the average for each replicate, colored by replicate to match the transparent dots. The black dot and lines represent the mean +/- SD.
