# Supplementary material for: Induction of Viral Mimicry Upon Loss of DHX9 and ADAR1 in Breast Cancer Cells
Source: Cancer Res Commun. 2024 Apr 4;4(4):986–1003. doi: 10.1158/2767-9764.CRC-23-0488 (PMC10993856; doi:10.1158/2767-9764.CRC-23-0488)
Supplement: Supplementary Figure 13 [file crc-23-0488-s15.pdf]

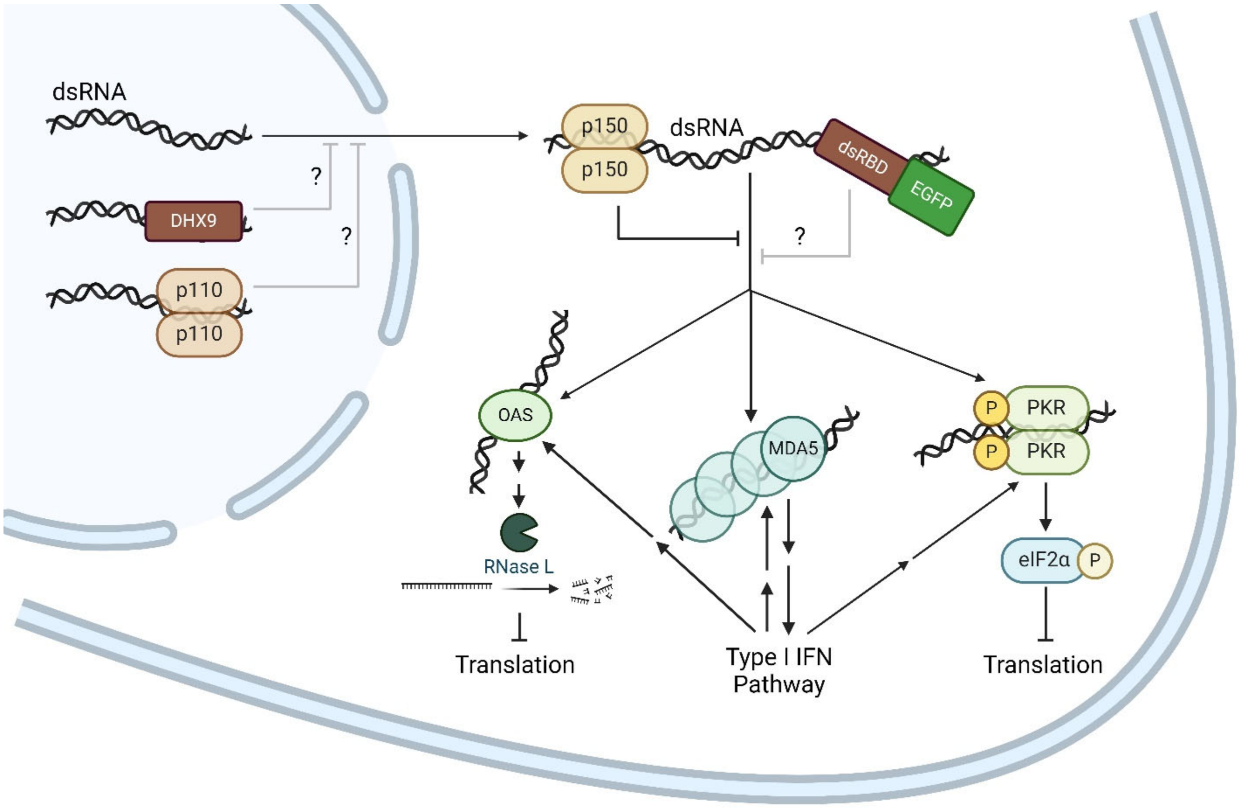

Figure S13:

A hypothetical model based on published data and the findings presented here. Gray lines marked with question marks are proposed and require further investigation. The protein cartoon labeled dsRBD EGFP represents the DHX9 N-terminal dsRBD-EGFP protein described in Figure 6. Created with BioRender.com
